# Spatiotemporal expression profile of novel and known small RNAs throughout rice plant development focussing on seed tissues

**DOI:** 10.1186/s12864-021-08264-z

**Published:** 2022-01-11

**Authors:** Anikó Meijer, Tim De Meyer, Klaas Vandepoele, Tina Kyndt

**Affiliations:** 1 Department of Biotechnology, Ghent University, Ghent, Belgium; 2 Department of Data Analysis and Mathematical Modelling, Ghent University, Ghent, Belgium; 3 Department of Plant Biotechnology and Bioinformatics, Ghent University, Ghent, Belgium; 4 VIB Center for Plant Systems Biology, Ghent, Belgium; 5 Bioinformatics Institute Ghent, Ghent University, Ghent, Belgium

**Keywords:** *Oryza sativa*, Embryo, Endosperm, Seed, Small RNA, siRNA, miRNA, tRFs, tsRNA, Diterpenoids

## Abstract

**Background:**

Small RNAs (sRNAs) regulate numerous plant processes directly related to yield, such as disease resistance and plant growth. To exploit this yield-regulating potential of sRNAs, the sRNA profile of one of the world’s most important staple crops – rice – was investigated throughout plant development using next-generation sequencing.

**Results:**

Root and leaves were investigated at both the vegetative and generative phase, and early-life sRNA expression was characterized in the embryo and endosperm. This led to the identification of 49,505 novel sRNAs and 5581 tRNA-derived sRNAs (tsRNAs). In all tissues, 24 nt small interfering RNAs (siRNAs) were highly expressed and associated with euchromatic, but not heterochromatic transposable elements. Twenty-one nt siRNAs deriving from genic regions in the endosperm were exceptionally highly expressed, mimicking previously reported expression levels of 24 nt siRNAs in younger endosperm samples. In rice embryos, sRNA content was highly diverse while tsRNAs were underrepresented, possibly due to snoRNA activity. Publicly available mRNA expression and DNA methylation profiles were used to identify putative siRNA targets in embryo and endosperm. These include multiple genes related to the plant hormones gibberellic acid and ethylene, and to seed phytoalexin and iron content.

**Conclusions:**

This work introduces multiple sRNAs as potential regulators of rice yield and quality, identifying them as possible targets for the continuous search to optimize rice production.

**Supplementary Information:**

The online version contains supplementary material available at 10.1186/s12864-021-08264-z.

## Background

The monocotyledonous model plant rice (*Oryza sativa*) is a staple crop for over half the world. With the growing human population, rice production will become even more important in the near future, challenging agronomists to find environmentally sustainable strategies to increase rice yield [1]. A thorough understanding of how biological processes such as plant development and disease resistance are controlled is key in achieving this goal.

Both plant development and reaction to (a)biotic stress are regulated by small non-coding RNAs (sRNAs) [2–5]. Classes of sRNAs in plants are microRNAs (miRNAs), small interfering RNAs (siRNAs) and transfer RNA (tRNA)-derived sRNAs (tsRNAs) [6, 7]. miRNAs and siRNAs are produced from hairpins or other double-stranded RNA precursors and cleaved by Dicer-like (DCL) enzymes to yield 20–24 nucleotide (nt) sRNAs. These 20-24 nt sRNAs are then incorporated into Argonaute (AGO) and guide AGO to their target by sequence complementarity. Post-transcriptional gene silencing is induced by target mRNA cleavage or translational repression [3]. Twenty-four nt siRNAs, on the other hand, mainly cause transcriptional repression through the plant-specific RNA-directed DNA methylation (RdDM) pathway, inducing de novo DNA methylation [8]. The different AGO proteins preferentially load sRNAs with specific 5′ end nucleotides, e.g. U in many miRNAs [9].

tsRNAs are generated after cleavage of tRNAs into smaller RNA fragments [7]. Based on their length, tsRNAs are classified as 14-30 nt tRNA fragments (tRFs) and 31-40 nt tRNA halves (tiRs). These two classes are further subdivided according to their position in the original tRNA: 5′ end of the mature tRNA (5tiR and tRF-5), 3′ end of the mature tRNA (3tiR and tRF-3) or the 3′ trailer of the primary tRNA sequence (tRF-1) [7]. Both tiRs and tRFs have been shown to regulate gene expression in eukaryotes, by interfering with translation or by association with AGO proteins, respectively [10–13].

sRNAs are crucial in the regulation of virtually all development- and stress-related processes throughout the plant’s lifecycle, such as germination, flowering, seed development, maintaining genomic integrity and resistance against numerous biotic and abiotic stress factors [14–17]. In the seed, for example, overexpression of MIR397 results in higher seed yield in both Arabidopsis and rice [16] and MIR398 is indispensable for correct cell patterning in Arabidopsis embryos and influences oil composition in *Brassica napa* seeds [18, 19]. In line with the role of specific sRNAs in different processes, the sRNA profile can be highly variable between tissues and plant life stages [16]. For example, certain 24 nt siRNAs in rice endosperm were shown to accumulate to much higher levels than any 24 nt siRNA in other tissues. Therefore, Rodrigues et al. (2013) [20] categorized these highly expressed loci into a new class termed siren (siRNA in endosperm).

As of November 2021, 12,059 miRNA and siRNA loci have been discovered in the dicotyledonous model plant Arabidopsis and at least 133,803 loci have been identified in rice [21–24]. In both species, 95% of these loci produce 24 nt siRNAs [21], which are well-known to target transposable elements (TEs) through the RdDM pathway [8]. Recent sRNA locus prediction by read clustering and subsequent locus classification contributed considerably to these available sRNA annotations [21]. For rice, 128 libraries were used, including 67 leaf/shoot samples and 33 root samples. However, with the exception of a single leaf sample, these were all obtained from young plants in the vegetative phase, i.e. before flower development. Also the economically important seed tissues were underrepresented with only 4 libraries from developing seeds: two grain samples, one embryo and one endosperm sample.

In this work we analysed the sRNA profile throughout the entire rice lifecycle. Roots and leaves were sampled at both the generative and the vegetative phase and early-life sRNA expression was studied in the embryo and endosperm. Not only expression of previously annotated miRNAs and siRNAs was investigated, but also new sRNA loci and tsRNAs were identified. Further, our data suggests the existence of 21 nt siren in the endosperm. Focusing on the economically important embryo and endosperm tissues led to the prediction of multiple siRNAs potentially involved in plant hormone regulation and/or signalling in rice seeds. These new insights increase our understanding of sRNA profiles in monocots and serve as a starting point for further research to improve rice yield and quality.

## Results

### sRNA profiling in different tissues expands the RNA landscape in rice

Next-generation small RNA sequencing was performed on multiple tissues and developmental stages of the rice cultivar Kitaake: root and leaves of plants in the vegetative phase (2.5 weeks old), root and leaf blades of plants in the reproductive phase (seed ripening stage) and the developing embryo and endosperm (sampled from plants in seed ripening stage). Mapping statistics can be found in Supplementary Table 2.

**Fig. 1:**
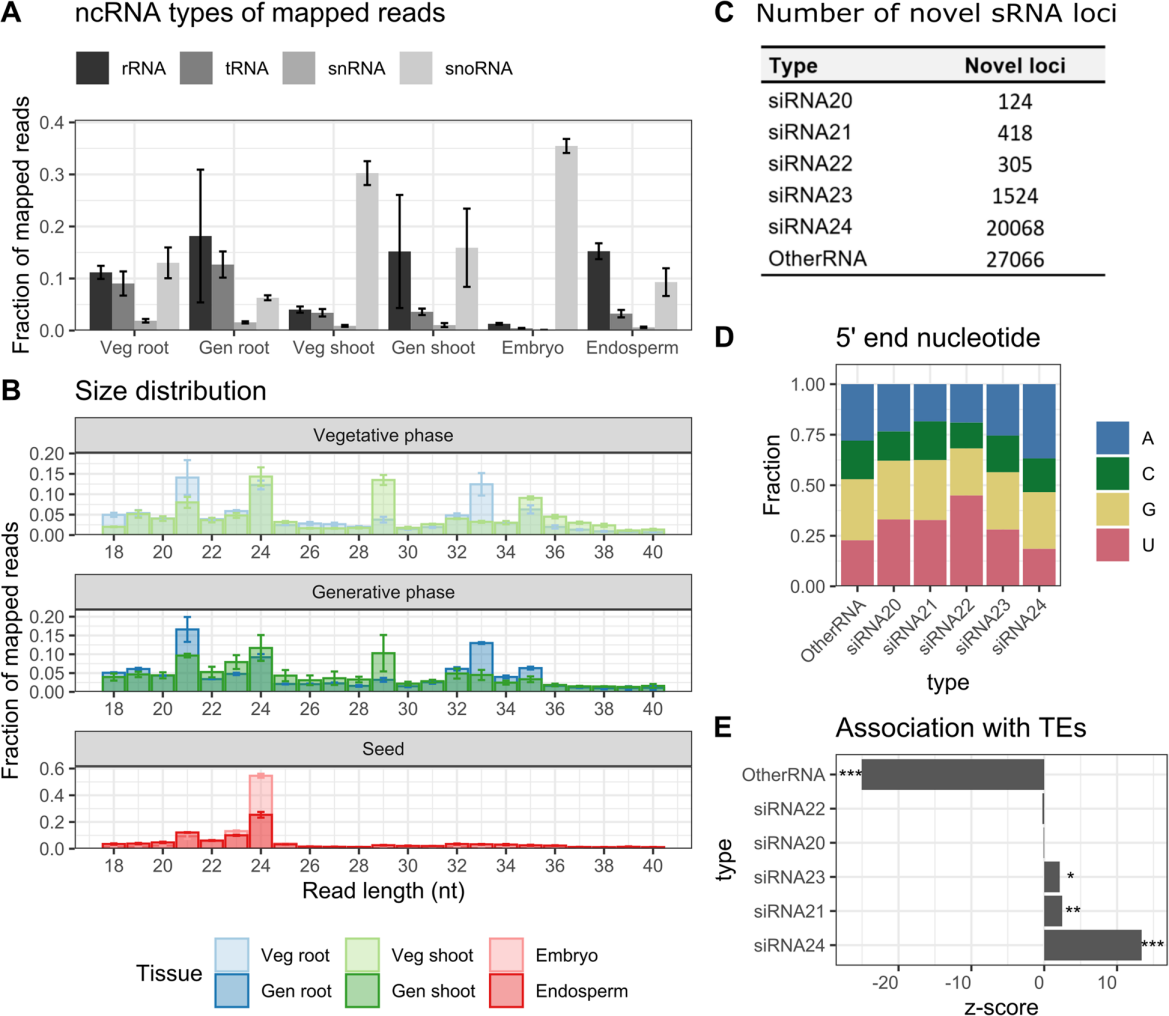
sRNA-seq read mapping on the rice genome and novel sRNA loci. **A** Fraction of mapped reads aligning to ribosomal RNA (rRNA), transfer RNA (tRNA), small nuclear RNA (snRNA) or small nucleolar RNA (snoRNA) loci. **B** Size distribution of 18-40 nt mapped reads. In all cases, error bars indicate the standard deviation. Veg = vegetative phase; Gen = generative phase. Veg shoot indicates leaves and gen shoot indicates leaf blades. **C** Number of novel loci for each sRNA type. Classification is based on the results of ShortStack cluster calling [25]. **D** Frequency of occurrence of each nucleotide at the 5′ end of novel sRNA loci. **E** Association of the different types of novel sRNA loci with TE regions in the rice genome. Permutation tests with 1000 permutations were performed to estimate the null distribution of sRNA loci randomly overlapping with the regions of interest, using regioneR. z-scores were calculated as the deviation of the actual number of overlaps from the number of overlaps under the null distribution, relative to the standard deviation. Stars indicate significant enrichment/depletion * = *p* < 0.05; ** = *p* < 0.01; *** = *p* < 0.001. In all panels siRNAx = locus mainly producing reads of length x; OtherRNA = loci where less than 80% of the reads are of 20-24 nt length, therefore considered as not processed by DCL enzymes and thus not classified as siRNA or miRNA

To study novel miRNAs and siRNAs, annotated ribosomal RNAs (rRNAs), tRNAs, small nuclear RNAs (snRNAs) and small nucleolar RNAs (snoRNAs) were removed from the data (Fig. 1A). 60–70% of the mapped reads remained. Notably, 35 and 30% of all mapped reads in the embryo and vegetative phase leaves mapped to snoRNA loci, respectively (Fig. 1A). All snoRNA-derived reads were longer than 75 nt, indicating that they are intact (Supplementary Fig. 1A). More than 99% of the snoRNAs expressed in the here-investigated samples belonged to the class of C/D-box snoRNAs, indicating that C/D box snoRNAs are highly expressed in the embryo and vegetative phase leaves.

The size distribution of reads not originating from rRNA, tRNA, snRNA or snoRNA loci revealed two peaks at 21 nt and 24 nt length, conform the predominant sizes generally reported in angiosperms (between 18 and 26 nt) [26] (Fig. 1B, unique sequences only: Supplementary Fig. 1B). Next to these two peaks, additional peaks at 29, 33 and 35 nt were observed. These were found to derive from tsRNAs and are further discussed below.

### Identification of novel TE-associated 24 nt siRNAs

Despite the many sRNA loci recently discovered in rice by Liu et al. (2017) (PLN24NT database) [23] and Lunardon et al. (2020) (Plant Small RNA Genes database) [21], 23 and 27% of the sRNA reads originated from unannotated loci in the generally understudied embryo and endosperm tissues, respectively (Supplementary Table 3). Therefore ShortStack [25, 27] was used to annotate novel sRNA genes based on our dataset.

All unannotated reads were pooled and 230,493 novel clusters were identified. After removing clusters overlapping with previously annotated sRNA loci [21, 23] and selecting those that were expressed in at least 2 samples (FPKM > 2), 49,505 novel sRNA loci were identified (available as supplementary material). To evaluate if these loci are potential miRNAs or siRNAs, ShortStack analyses the read lengths in each cluster. If for a specific cluster 80% of the reads was situated between 20 and 24 nt in length these reads were assumed to originate from DCL processing. Loci that did not match these criteria were classified as ‘OtherRNA’. More details on sRNA classification are explained in [25].

45% of the newly identified clusters were predicted to be DCL-processed, of which 89% predominantly produced 24 nt siRNAs (siRNA24). Other classes of siRNAs (siRNA20–23) were barely represented (Fig. 1C). As expected, the dominant 5′ end nucleotide of the different sRNA types was U for novel siRNA20–22 (34–51%), while this was A for 37% of the novel siRNA24 (Fig. 1D) [9].

In plants, TEs are silenced by 24 nt siRNAs through the RdDM pathway [8]. To study the association between the newly identified loci and TEs, the R bioconductor regioneR package was used (see Methods). siRNA24 loci were highly enriched in TE regions, with a z-score of 13.4 (Fig. 1E). Interestingly, the class of OtherRNAs was significantly depleted in TEs (z-score = − 25.1) (Fig. 1E). Together, this suggests that a substantial number of the newly identified siRNA24 loci are involved in TE silencing, while the functionally still uncharacterized OtherRNAs do not seem to play a role in this process.

Similarly, novel siRNA 22–24 and OtherRNA were significantly depleted in 1 kb upstream, the gene body and 1 kb downstream of annotated protein-coding genes (Supplementary Fig. 2), indicating that the majority of these novel loci may not be directly involved in gene expression regulation.

### Identification of three seed-specific miRNAs

Under a stringent expression cut-off (FPKM > 2 in all biological replicates), 12 expressed miRNAs were found in our dataset. Of these, 5 were shared between all tissues, including a miR1846 family member (Supplementary Table 4). This miR1846 was highly expressed throughout the rice lifecycle in root, shoot and endosperm (FPKM ranging between 351 and 33) and moderately in the embryo (FPKM 9.8), indicating that it might play an important role in cells of all organs and developmental stages. Additionally, 1 embryo-specific miRNA of the miR394 family and 1 endosperm-specific miRNA of an unknown family were identified. Lastly, expression of osa-MIR169o was detected in embryo and endosperm but not in any other investigated tissue, pointing to a seed-specific role for this miRNA.

### siRNA24 are highly expressed in all plant organs and function in DNA transposon, but not LTR silencing

**Fig. 2:**
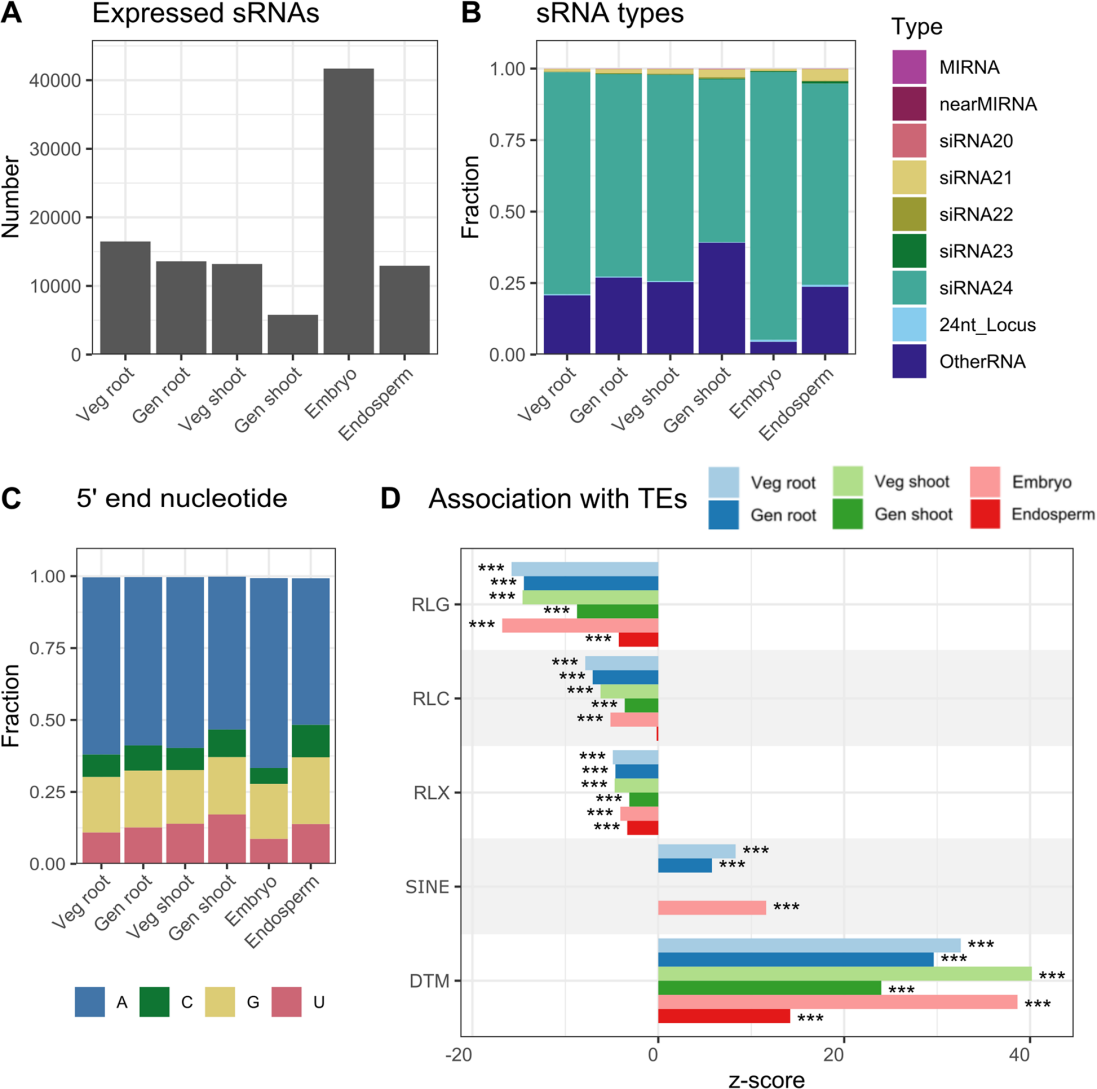
Small RNA expression in various rice tissues. **A** Number of expressed sRNAs detected in the 6 studied tissues. sRNA genes were considered expressed when they had an FPKM value higher than 2 for each of the biological replicates. **B** Types of expressed sRNA genes. MiRNA = loci producing miRNAs; nearMiRNA = loci predicted to be miRNA loci, but lacking the miRNA* passenger strand [21]; siRNAx = loci mainly producing siRNAs of length x; 24nt_Locus = loci from the pln24nt database. These were classified separately because no information is available on the predominant sRNA size expressed from these loci; OtherRNA = loci where less than 80% of the reads are 20-24 nt long and therefore considered not processed by DCL enzymes. **C** Frequency of occurrence of each nucleotide at the 5′ end of expressed sRNA loci in each tissue. **D** Association of expressed sRNAs in the 6 tissues/time points with 5 TE families. Permutation tests were conducted as described in Figure 2. Bars were not shown if the permutation null distribution did not resemble a normal distribution. DTM = DNA transposon mutator, RSU = retroelement short interspersed nuclear element; RLC = retroelement long tandem repeat Copia; RLG = retroelement long tandem repeat Gypsi; RLX = retroelement long tandem repeat ‘Unknown’. In all panels: Veg = vegetative phase; Gen = generative phase. Veg shoot indicates leaves and gen shoot indicates leaf blades

To uncover differences in the sRNA profile between vegetative, generative and seed tissues, expression of previously annotated and novel sRNAs was studied for each tissue and developmental stage. Strikingly, in the embryo three times as many sRNAs were expressed in comparison to the other tissues (i.e. FPKM > 2 in all biological replicates) (Fig. 2A). Furthermore, sRNA expression seems to decrease when comparing vegetative to generative plants, both in leaves and in roots (Fig. 2A).

10–20% of the expressed sRNAs originated from novel loci, emphasizing the importance of identifying novel sRNA genes, as many of them are still unannotated (Supplementary Fig. 3). The majority of the expressed sRNAs were siRNA24 loci, especially in the embryo where they made up 94% of all expressed sRNAs (Fig. 2B). As expected for 24 nt siRNA-dominated expression profiles, the majority of the expressed sRNAs had a 5′ end A in all tissues (Fig. 2C). Taken together, this indicates that siRNAs of length 24 are highly abundant in all studied plant organs.

To investigate the role of these sRNAs in TE silencing, their association with different TE families was evaluated using regioneR (see Methods). In all tissues, we found significant overlap between sRNAs and DNA transposon mutator (DTM), with the highest association in embryos and vegetative phase shoots. Additionally, in the embryo and root short interspersed nuclear elements (SINEs) were also enriched for sRNAs. In contrast, all three long tandem repeat (LTR) families (Copia, Gypsy and “unknown”) are depleted for expressed sRNAs in almost all tissues. For all TE families, the weakest association with sRNAs was always found in the endosperm (Fig. 2D). Together this illustrates the importance of sRNAs in DTM and SINE, but not LTR silencing in all studied tissues.

### Rice tsRNAs mostly derive from the 5′ end of mature tRNAs and are relatively scarce in the embryo

In the size distribution of mapped reads, three previously undescribed peaks were observed: 29 nt in leaf (blade) samples and 33 and 35 nt in roots (Fig. 1B). sRNAs that fall within these size ranges are the recently discovered tsRNAs [7]. Similar to miRNAs and siRNAs they can also silence gene expression, although the functional mechanisms are still poorly understood [7].

A BLAST database was built from mature tRNA sequences (including the 3′ CCA) and from the tRNA sequences flanked by their 40 bp upstream and 40 bp downstream regions to mimic primary tRNAs (as in Gupta et al., 2018 [28]). Sizes of root and leaf (blade) reads originating from these tRNA regions indeed showed peaks at 29, 33 and 35 nt. Furthermore, virtually none of these reads were longer than 38 nt, indicating that these were not intact tRNAs (Supplementary Fig. 5). To study if these reads could be tsRNAs or rather random tRNA breakdown products, we compared the positions of read start and read end to the rice tRNA transcripts and found peaks at the first base (5tiR and tRF-5) and the last base of the tRNA (3tiR and tRF-3), respectively (Supplementary Fig. 6). For random breakdown, a more even distribution would be expected, indicating that the tRNA-derived reads found here are likely to be tsRNAs.

For independent validation, the presence of 2 selected 5tiR tsRNAs (from PheGAA and GluTTC) and 2 tRF-5 tsRNAs (from AlaCGC and ArgACG) was confirmed with stem-loop RT-PCR in root and leaves (Supplementary Fig. 10). In total 4334 tRFs and 1247 tiRs were detected (available as supplementary material). BLAST comparison to the plant tRF database [28] and tsRBase [29], revealed that 4115 of these tsRNAs had not been identified in rice before.

**Fig. 3:**
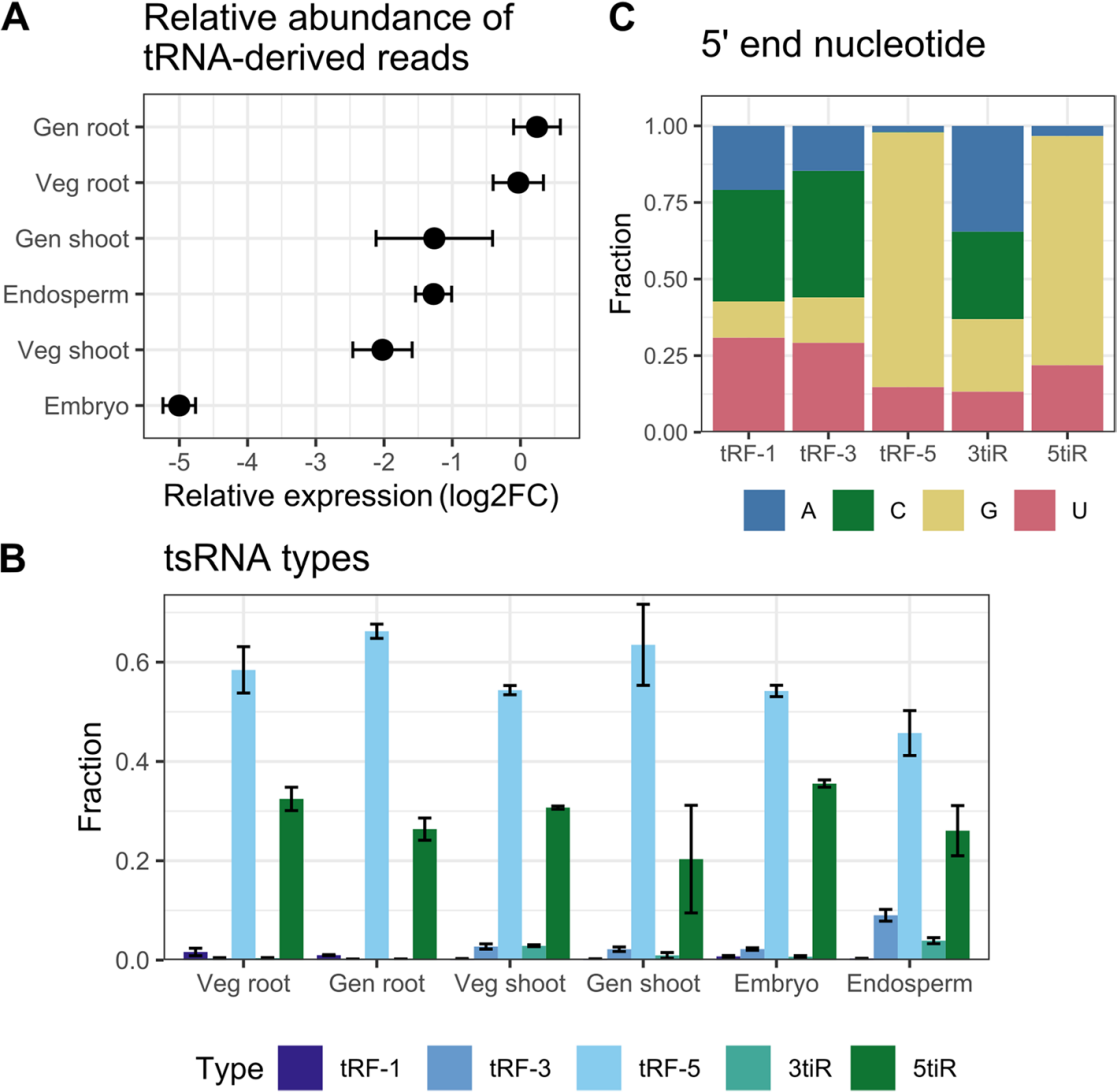
tRNA-derived sRNAs in rice. **A** Relative expression of 14-40 nt tRNA-derived reads, normalized to 4 reference genes and expressed as log2 fold change (log2FC) compared to vegetative phase roots. For details see Methods. **B** Classification of tRNA-derived reads based on their length and position in the tRNA gene, as described in Kumar et al. (2016) [7]. tRF-1: reads of 14-30 nt aligning in the 3′ trailer; tRF-3: reads of 14-30 nt with alignment ending in the last 3′ base of the tRNA; tRF-5: reads of 14-30 nt with alignment starting at the first 5′ base of the tRNA; 3tiR: reads of 31-40 nt with alignment ending in the last 3′ base of the tRNA; 5tiR: reads of 31-40 nt with alignment starting at the first 5′ base of the tRNA. **C** Frequency of occurrence of each nucleotide at the 5′ end of the different tsRNA types. In panel **A** and **B**, error bars indicate the standard deviation. Veg = vegetative phase; Gen = generative phase. Veg shoot indicates leaves and gen shoot indicates leaf blades

To compare the relative abundance of tsRNAs in the different tissues, tRNA-derived read counts in each sample were normalized to four here-identified stably expressed ‘reference’ sRNAs (osa-b1.0r1–56,010, osa-b1.0r1–70,282, osa-b1.0r1–75,354, osa-b1.0r1–87,012) (see Methods). Strikingly, the tsRNA-derived reads were less abundant in the embryo sRNA dataset compared with the other tissues (log2FC = − 5 in comparison to vegetative phase root) (Fig. 3A). In all samples 70–94% of all tRNA-derived reads were tRF-5 or 5tiR (Fig. 3B). Considering 5′ ends, in tRF-5 s and 5tiRs the majority started with G (83 and 75%, respectively) (Fig. 3C). Taken together, we conclude that tsRNAs are relatively scarce in the embryo and that in all tissues 5′-derived tsRNAs starting with G dominate the tsRNA profile. The latter has been generally observed in plants before [12, 13, 28, 30, 31], illustrating the robustness of our data.

C/D box snoRNAs – highly expressed in the embryo (Fig. 1A) – are known to methylate RNA [32], a process which protects the tRNA from cleavage [10]. Indeed, we found a significant negative correlation between the number of tsRNA reads and C/D box snoRNA reads in each sample (*p* < 0.001, Kendall rank correlation test) (Supplementary Fig. 7). This indicates that snoRNA activity might prevent the cleavage of tRNAs into tsRNAs in rice embryos.

### Tissue-specific siRNA expression indicates that many seed-specific siRNAs are 24 nt and shared between embryo and endosperm

**Fig. 4:**
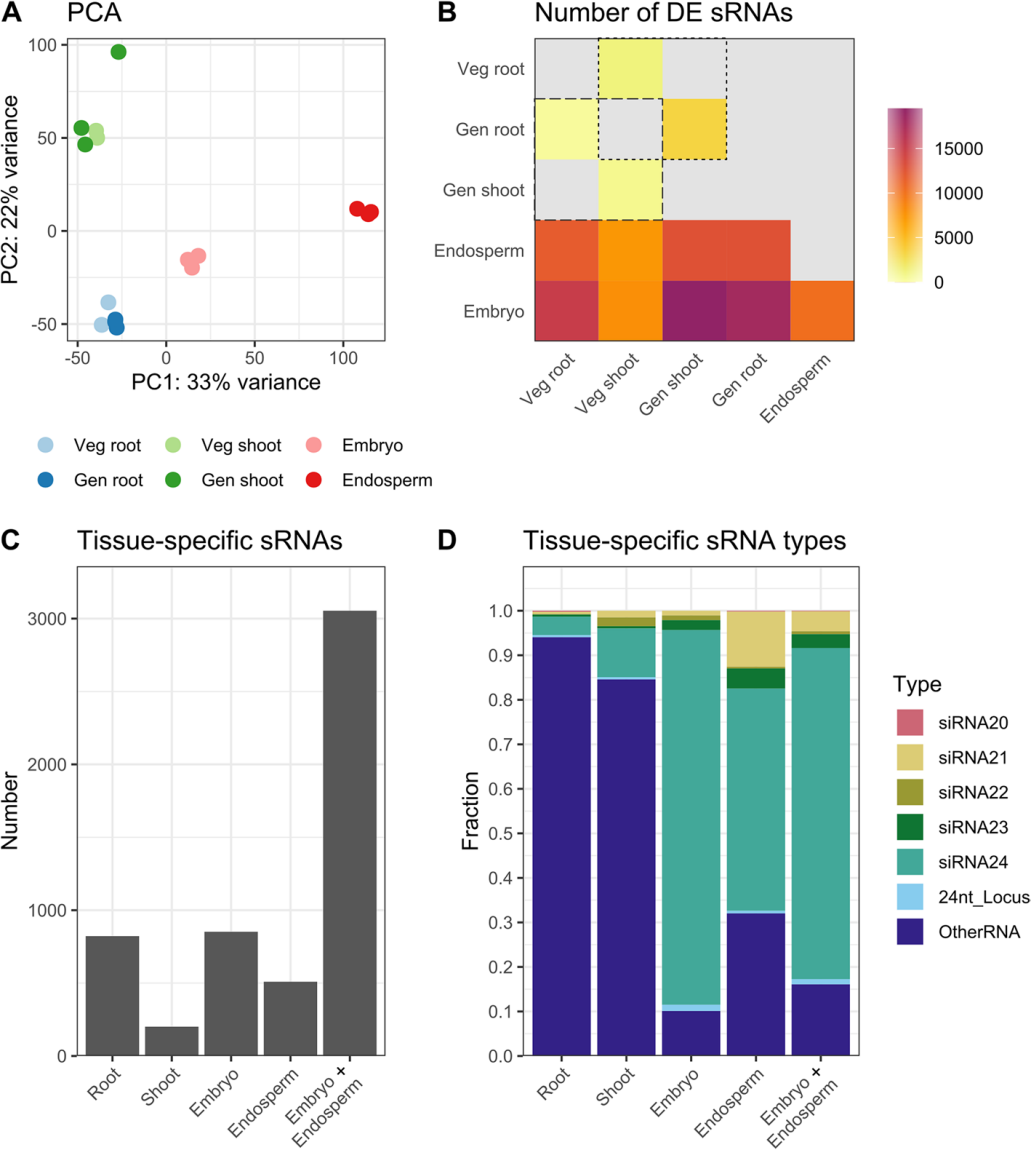
Tissue-specific sRNA expression. **A** Principal component analysis (PCA) of sRNA expression in the six studied tissues/time points. The principal components were calculated based on the top 500 most variable sRNA genes between samples. **B** Number of differentially expressed genes (DEGs) resulting from pairwise differential expression analysis between tissues. Dashed square: comparisons between the same organ at different developmental stages; dotted square: comparisons between different organs at the same time point. In **A** and **B** Veg = vegetative phase; Gen = generative phase. Veg shoot indicates leaves and gen shoot indicates leaf blades. **C** Number of tissue-specific sRNAs. Tissue-specific expression was defined as having a FPKM higher than two in the tissue of interest and a read count of zero in all other tissues. Root or leaf (blade) samples at vegetative and generative stage were combined. The group ‘embryo + endosperm’ indicates sRNAs specifically expressed in embryo and/or endosperm. **D** Classification of tissue-specific sRNA genes. siRNAx = loci mainly producing siRNAs of length x; 24nt_Locus = loci from the pln24nt database. These were classified separately because no information is available on the predominant sRNA size expressed from these loci; OtherRNA = loci where less than 80% of the reads are 20-24 nt long, therefore considered as not processed by DCL enzymes

Principal component analysis (PCA) on the sRNA profiles of all samples showed that sRNA expression was more divergent between organs than between developmental phases of the same organ (Fig. 4A). These trends were also confirmed by the number of differentially expressed (DE) sRNA genes between groups (Fig. 4B). Additionally, the difference in sRNA expression between root and leaf (blade) appears greater at the generative than at the vegetative phase (Fig. 4B). Together, this indicates that the sRNA profile is more closely associated with the studied plant organ than the developmental phase.

sRNAs featuring plant organ or developmental phase specific expression are likely to play a tissue- or time-specific role. As differences between plant organs were considerably larger than between developmental phases (Fig. 4A-B), tissue-specific sRNAs were analysed regardless of the latter. They were defined as genes expressed in the tissue of interest, but with a read count of zero in all other samples. Next to studying embryo and endosperm separately, an additional group “embryo + endosperm” was considered with sRNAs that were specifically expressed in embryo, endosperm or both.

More than 3000 embryo + endosperm-specific sRNAs were discovered, while this was only 852 and 509 for embryo and endosperm alone, respectively (Fig. 4C). Thus, the majority of the embryo and/or endosperm-specific sRNA genes are expressed in both seed tissues. Comparing the expression levels of these common genes revealed quite distinct expression between both tissues (Supplementary Fig. 4). Two hundred seventy-five common genes were significantly DE, of which three were overexpressed in the embryo and 272 in the endosperm, when compared to the other tissue. Notably, 74% of all seed-specific genes are siRNA24 loci, while more than 80% of root or leaf-specific sRNAs are OtherRNAs (Fig. 4D). In short, our data shows that seed-specific siRNA are mostly 24 nt long and common between embryo and endosperm, albeit with generally higher expression levels in the latter.

### Association of seed siRNAs with coding genes suggests that 24 nt siRNAs suppress gene expression from the terminator

Despite the fact that the seed is the most important rice product in terms of human consumption, the sRNA profile of embryo and endosperm has not been studied extensively before. To relate sRNA expression to the regulation of protein-coding genes, publicly available expression data of one embryo and two endosperm samples was retrieved from the sequence read archive (SRA) [33]. This data was used to classify protein-coding genes into highly expressed genes (average FPKM > 10), medium expressed genes (average FPKM between 2 and 10), lowly expressed genes (average FPKM between 2 and 0) and non-expressed genes (average FPKM = 0) in both tissues.

**Fig. 5:**
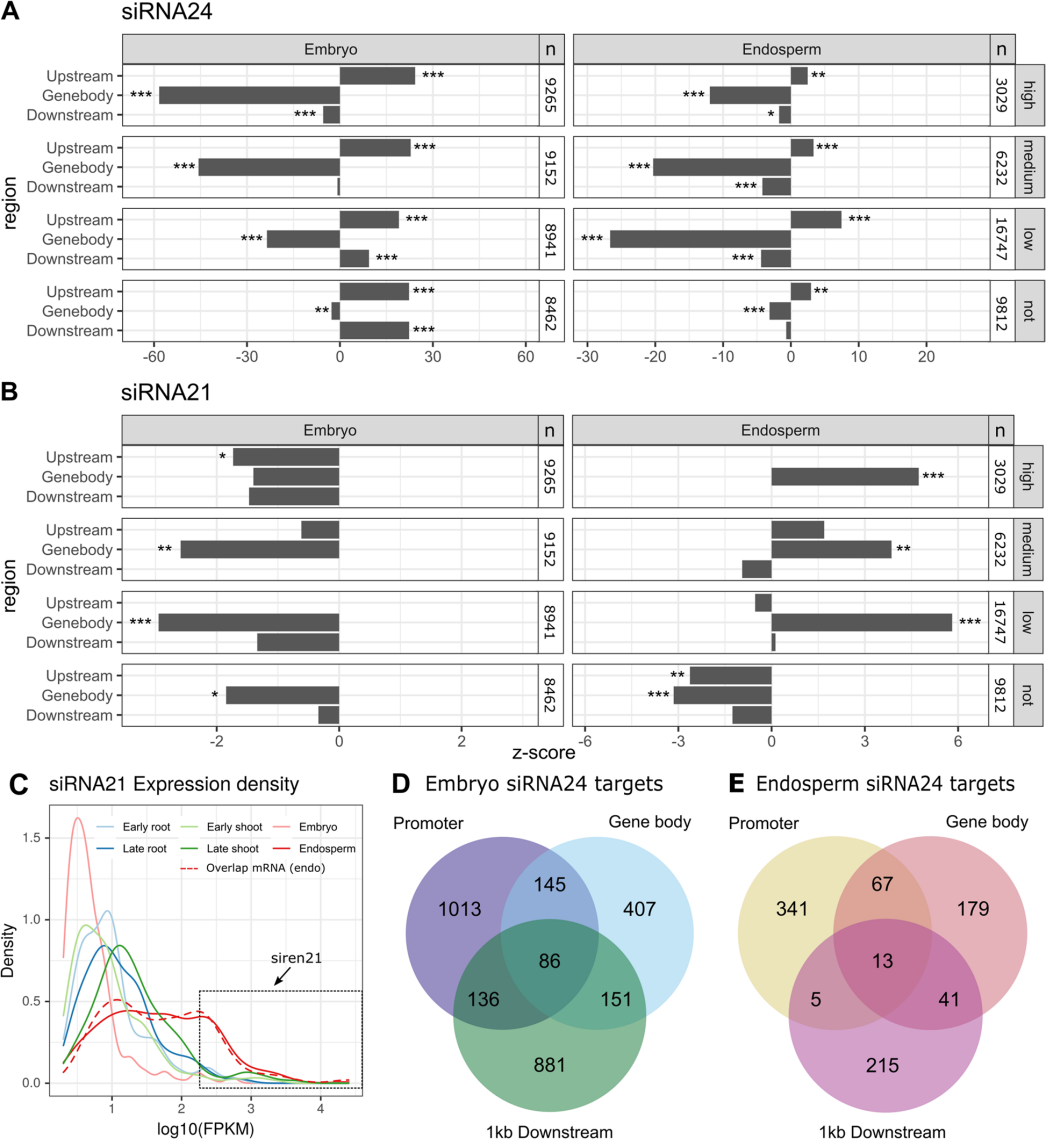
siRNA-regulated genes in rice embryo and endosperm. **A-B** Overlap between siRNA24 loci (**A**) or siRNA21 loci (**B**) in embryo and endosperm, and protein-coding genes in their 1 kb upstream, gene body or 1 kb downstream regions. For both tissues, all coding genes were categorized based on their expression level in the corresponding tissue: high (FPKM > 10), medium (FPKM between 10 and 2), low (FPKM between 2 and 0) or not expressed (FPKM = 0). n indicates the number of protein-coding genes in each group. Permutation tests were conducted as described in Fig. 2. Data where the null distribution did not resemble a normal distribution are not shown. **C** FPKM density of expressed siRNA21 in all tissues and for siRNA21 overlapping expressed protein-coding genes in the endosperm (red dashed line). The box indicates the FPKM cut-off for the exceptionally highly expressed siren21 loci. **D-E** Number of predicted targets of siRNA24 in the embryo (**D**) and endosperm (**E**), overlapping with the promoter (1 kb upstream), the gene body or 1 kb downstream regions of coding genes

Next, the overlap of expressed siRNA21 or siRNA24 with these protein-coding genes was investigated using regioneR (see Methods). siRNA24 loci were significantly depleted in the gene body and enriched in 1 kb upstream regions of protein-coding genes (Fig. 5A). In the embryo, they were also significantly enriched in the downstream regions of lowly or non-expressed genes, with z-scores of 9.4 and 22.3, respectively. This indicates that the expression of siRNA24 in the terminator region of protein-coding genes might induce a decrease in mRNA abundance in the embryo.

Endosperm-expressed siRNA21 were significantly enriched in the gene body of expressed protein-coding genes, regardless of the expression level (low, medium or high) (Fig. 5B). In contrast, non-expressed genes were significantly depleted for siRNA21s in their gene body (z-score = − 3.2) or 1 kb upstream regions (z-score = − 2.6) (Fig. 5B). This indicates that siRNAs of length 21 nt arise from expressed mRNAs in the endosperm. Secondary mRNA-derived siRNAs are typically produced in a phasing pattern. However, there was considerably less phasing among siRNA21 loci overlapping mRNAs, compared to all expressed siRNA21 in the endosperm (10.5% vs 20.3%, respectively, chisquared *p* = 0.002), rejecting the hypothesis that these siRNA21 loci in the endosperm are secondary siRNAs.

In the embryo there was no such enrichment of gene body siRNAs (Fig. 5B), indicating functional divergence of 21 nt siRNA between both tissues. To further consolidate this hypothesis, we compared genes with an endosperm-expressed siRNA21 locus in their gene body with genes carrying and embryo-expressed siRNA21 in their promoter, gene body or downstream regions. Of the 198 and 103 genes, respectively, only 35 were common. This indicates that these gene body derived siRNA21 in the endosperm might target a different set of genes, which is not regulated by siRNA21 in the embryo.

### Solid stage endosperm contains 21 nt sirens

Rodrigues et al. (2013) [20] identified sirens as all highly expressed 24 nt siRNA in immature (milky) endosperm. However, siRNA24 expression density in solid stage endosperm was not clearly shifted towards higher FPKM values (Supplementary Fig. 8). Surprisingly, instead siRNA21 expression density was skewed to higher values in the endosperm (Fig. 5C). These highly expressed loci (FPKM > 10^2.25^) were termed siren21 (Supplementary Table 5). Next, we investigated if the non-phased siRNA21 from expressed genes described above could be categorized as siren21. Indeed, siRNA21 overlapping endosperm-expressed mRNAs were skewed towards high expression values (Fig. 5C). Of all 108 siren21 we identified, 57% overlapped with the gene body of expressed genes. This is a significant enrichment compared to non-siren siRNA21 (chisquared *p* = 0.04). Therefore, we conclude that siren21 are expressed in solid stage rice endosperm and at least partly originate from protein-coding genes in a non-phased pattern.

### Putative siRNA24 targets in the embryo potentially regulate seed iron, hormone and phytoalexin content

To elucidate which genes are potentially regulated by siRNAs in the embryo and endosperm, we searched for protein-coding genes that contain embryo- or endosperm-expressed siRNA24 in their promoter (1 kb upstream), gene body or 1 kb downstream regions. Since 24 nt siRNA targets are expected to be methylated and to have low expression levels, siRNA24-overlapping genes were compared to publicly available expression [33] and DNA-methylation data [34] in rice embryo and endosperm. Genes were only selected as potential targets when the corresponding region (promoter, gene body or 1 kb downstream) was heavily methylated (methylation levels in the upper quantile of corresponding tissue, i.e. > 20% for embryo and > 12% for endosperm) and when they were lowly to non-expressed (FPKM < 2). For promoter, gene body and 1 kb downstream, this led to 1380, 789 and 1254 potential targets in the embryo, and 426, 300 and 274 potential targets in the endosperm, respectively (Fig. 5D-E) (Supplementary Tables 6–7).

**Fig. 6:**
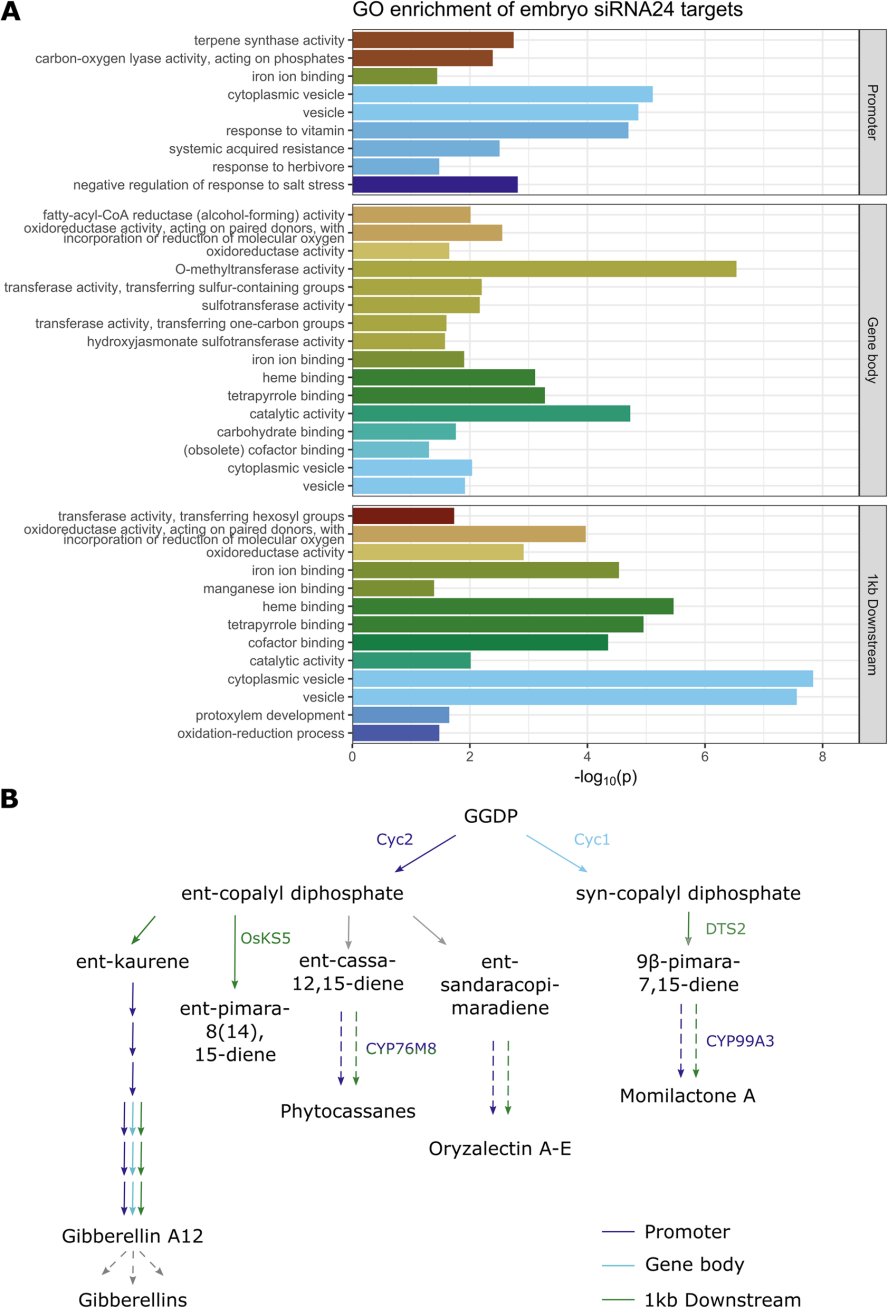
Function of putative siRNA24 targets in the embryo. **A** Gene ontology enrichment analysis, carried out with Monocots PLAZA 4.5 [35]. *P*-values were Bonferroni-corrected for multiple testing and the results were summarized using REVIGO [36]. Bars with the same colour indicate related GO terms. **B** Biosynthesis of diterpenoids, as annotated in OryzaCyc (v. 7.0). Colours indicate expression of an overlapping siRNA24 in the embryo, for promoter (1 kb upstream), gene body and 1 kb downstream regions. Rice genes for which enzymatic activity has been confirmed experimentally are shown next to the arrows. Dashed arrows indicate multiple reactions, catalysed by a single or multiple enzymes. A detailed description of these enzymes and their overlapping siRNA24 is given in Supplementary Table 9. GGDP = geranyl geranyl diphosphate; Cyc = cyclase, OsKS5 = Ent-kaurene synthase 5, CYP = Cytochrome P450, DTS2 = 9b-pimara-7,15-diene synthase

Comparing expression profiles of siRNA24 loci in protein-coding genes, expression in the promoter was distinct from gene body or downstream in both embryo and endosperm. Further, siRNA24 expression is globally higher in the endosperm than in the embryo (Supplementary Fig. 9). Among endosperm siRNA targets, we found one enriched GO term: sulfotransferase activity (GO:0008146, log2 enrichment fold 4.16, Bonferroni-corrected *p*-value 0.016) in the gene body targets. Also in the embryo three sulfotransferase-related terms were significantly enriched among putative targets with gene body overlapping siRNA24 (Fig. 6A, Supplementary Table 8), indicating siRNA-mediated regulation of sulfotransferase activity in both tissues.

Among the potential siRNA targets in the embryo, gene ontology (GO) analysis revealed significantly enriched (Bonferroni-adjusted *p* < 0.05) terms related to iron ion and/or heme binding, as well as ‘cytoplasmic vesicle’ (Fig. 6A, Supplementary Table 8). Among iron ion binding proteins, there were 21 encoding a cytochrome P450 protein that overlapped with an siRNA24 in their promoter. Together, this suggests a role for 24 nt siRNAs in seed iron content and vesicle formation.

Also, terpene synthase activity was significantly enriched among potential embryo targets with siRNA24 in their promoters (GO:0010333, *p* = 0.002). Mapping potential siRNA-regulated genes to the metabolic pathways in OryzaCyc [37] suggested involvement of embryo siRNAs in the regulation of two classes of diterpenoids: gibberellic acid (GA) and phytoalexins. Eleven embryo 24 nt siRNAs putatively target almost every characterized step in the biosynthesis of gibberellin A12 (a branch point leading to a spectrum of active and inactive forms of GA), of the known phytoalexin precursors and of the phyotoalexins oryzalectin A-E, momilactone A and phytocassanes. (Fig. 6B, Supplementary Table 9). Both GA and phytoalexins are synthesized from the common precursor geranylgeranyl diphosphate (GGDP), which is then converted into either syn- or ent-copalyl diphosphate, leading to GA, oryzalectin A-E and phytocassanes or momilactone A, respectively (Fig. 6B). Our data suggests that both the “syn” and “ent” branches might be controlled by 24 nt siRNA in the rice embryo, as multiple genes in both pathways were identified as potential targets. Especially in the gene body of Cyc1 (Cyclase 1, GGDP to syn-copalyl diphosphate conversion) two siRNA24 are highly expressed (FPKM 7.07–7.09). The same is true for the downstream genes *CYP99A3* (Cytochrome P450 99A3) and *CYP76M8,* where highly expressed siRNA24 were found in the promoter and downstream region, respectively (FPKM 6.64 and 5.94, respectively) (Fig. 6B, Supplementary Table 9).

Lastly, according to MapMap annotation, multiple siRNA24 were potentially targeting genes involved in ethylene (ET) regulation and response, in both embryo (41 siRNAs) and endosperm (8 siRNAs) (Supplementary Table 10). Three siRNA24 loci were relatively highly expressed in both embryo and endosperm (FPKM 3.26–9.65) and potentially target 3 genes encoding AP2 domain containing proteins, involved in ET signalling (Os05g0473300, Os08g0360800 and Os10g0390800) (Supplementary Table 10). Together this data points towards a putative function of embryo 24 nt siRNAs in the regulation of ET content and response, although more research is required to validate these predictions.

## Discussion

sRNAs are important regulators of plant growth, development, stress tolerance and yield [38]. We present the sRNA expression profile of various rice tissues at different developmental phases: root and leaves at the vegetative phase, root and leaf blades at the generative phase, the embryo and the endosperm. By combining these well-studied and less studied tissues from different developmental stages, 49,505 novel small RNA genes were predicted in rice. We also identified 4334 tRFs and 1247 tiRs. Four thousand one hundred fifteen of these did not match any rice sequences in the plant tRF [28] and tsRBase [29] databases. Therefore, this work proves a valuable addition to sRNA annotation in rice at various developmental stages.

Seed sRNA size distribution seems to be conserved among angiosperms [16]. Also in gymnosperms embryo profiles are dominated by 24 nt siRNAs, especially at later stages. However, in gymnosperm non-seed tissues barely any 24 nt siRNAs were detected, indicating a major difference between both clades [39, 40].

The number of expressed sRNA loci was three to seven times greater in the embryo compared to other tissues. Generally, we observed an age-dependent decrease in sRNA expression (Fig. 2A). Cells of the developing embryo are dividing rapidly and in Arabidopsis embryos this has been suggested to allow increased expression of TE-derived 24 nt siRNAs [41]. Due to chromatin decondensation following fertilization and consecutive condensation and decondensation in rapidly dividing cells, POL IV gains access to otherwise suppressed TE regions allowing for increased 24 nt siRNA production [41]. While a peak in heterochromatic TEs is observed in early embryos, euchromatic TEs are expressed throughout embryo development. Both contribute to elevated 24 nt siRNA levels [41]. In maturing rice embryos, we found significant enrichment of sRNAs from euchromatic (DTM and SINE), but not heterochromatic (LTR Gypsy and Copia) TEs, conform the Arabidopsis model. Further profiling of rice embryos in different developmental stages is required to confirm this similarity.

In contrast to siRNAs, the embryo contains 8–32 times less tsRNAs than the other tissues under study (Fig. 3A). The RNAses responsible for tsRNA production have been identified in Arabidopsis as the RNAse T2 enzymes AtRNS1–3 [42]. RNS expression levels correlate well with tsRNA abundance in Arabidopsis upon phosphate starvation [42, 43]. In Rice, there are 8 RNS homologues, of which OsRNS2 and OsRNS6 have high sequence similarity to AtRNS2 and OsRNS3 is highly similar to both AtRNS1 and AtRNS3 (Clustal omega protein sequence alignment). The expression of these 3 OsRNS proteins decreases throughout embryo development [44, 45]. Thus, to understand the distinct tsRNA profile in the embryo it might be interesting to validate the role of *OsRNS* genes in tsRNA production and to possibly identify RNS activity as the driver behind tsRNA levels in the different rice tissues.

Additionally, we have shown that embryos possess high numbers of C/D box snoRNAs (35% of all reads) (Fig. 1A). C/D box snoRNAs are mainly involved in the processing of pre-rRNA to mature rRNA by 2′-O-ribose methylation [32], but in human cells evidence was found for a snoRNA inhibiting 3tiR formation by ribose methylation of the tRNA cleavage site [46]. Our data similarly revealed a significant negative correlation between the expression of C/D box snoRNAs and tsRNAs (Supplementary Fig. 7), leading to a hypothesis where snoRNAs are involved as one of the regulators of tsRNA production in rice.

Sirens are exceptionally highly expressed siRNA24 loci in the endosperm or ovules [20, 47]. To investigate their presence in solid stage rice endosperm, the expression density of siRNA24 was calculated. Contrasting earlier observations in immature (milky) endosperm [20], there was no clear increased expression of siRNA24 in our endosperm data set (Supplementary Fig. 8). Surprisingly, when analysing siRNA21 loci instead, there was a clear increase of expression level in the endosperm compared to other tissues (Fig. 5C). A possible cause for this discrepancy might be the different developmental stage of the endosperm: Rodrigues et al. (2013) [20] used milky stage endosperm while we used more mature, solid endosperm for our analyses. Therefore, we hypothesize that siren length varies throughout endosperm development. Early on they are mostly 24 nt in length (siren24), while in solid endosperm the majority is 21 nt in length (siren21).

The majority of these siren21 originate from the gene body of endosperm-expressed genes (Fig. 5B). Thus, they arise either from mRNA cleavage or from actively transcribed loci. As they are not produced in a phasing pattern, they are unlikely to be secondary siRNAs. Also siren24 in *Brassica rapa* ovules are unphased and overlap considerably with genic regions [47], indicating that all siren species might be primary siRNAs transcribed from protein-coding regions.

Focussing on the better-characterized miRNAs, one embryo-specific miRNA was identified (osa-b1.0r1–60,176, FPKM 5.84). This miRNA is classified as a miR394, a family found in both mono- and dicotyledonous plants. In Arabidopsis embryos, miR394 plays a role in shoot apical meristem (SAM) formation by targeting F-box protein AtLCR (LEAF CURLING RESPONSIVENESS, AT1G27340) [18]. Also on *Brassica napus*, miR394 targets BnLCR regulating seed development and oil content [19]. In rice, OsLC4 (LEAF INCLINCATION 4, Os01g0923900) is a homologue of LCR and has very high sequence complementarity to the mature miRNA, indicating that it might play a similar role in monocotyledonous embryos.

Another miRNA, osa-MIR169o, was identified in the embryo (FPKM 5.86) and endosperm (FPKM 39.17), but in none of the other tissues. This miRNA was found previously in salt-stressed rice and Arabidopsis seedlings and was hypothesized to be ABA-responsive [48]. It targets cleavage of NF-YA, one of three subunits of NF-Y transcription factors [48]. In the seed, the balance between ABA and GA regulates seed dormancy and germination. One of the regulators in this balance is an NF-Y complex containing NF-YC3, 4 or 9 [49]. Possibly, osa-MIR169o is regulated by ABA and in turn regulates seed dormancy through NF-Y, adding another layer to the complex regulation of ABA/GA levels in the seed. Further experiments, such as mutant studies, are required to confirm this hypothesis.

For functional prediction of the less-characterized siRNA24, we compared their expression with publicly available mRNA expression and DNA methylation in embryo and endosperm to search for potential targets. In the embryo, predicted siRNA24 targets were enriched for genes with functions related to iron ion or heme binding (Fig. 6, Supplementary Table 8). In rice plants, iron is and concentrated in the scutellum – sampled here as part of the embryo – and indispensable for embryo development [50]. Our predictions indicate siRNA24-mediated regulation of seed iron levels. If this would be further experimentally confirmed, these sRNAs might be a possible target for biofortification strategies to combat iron deficiency-induced anemia, especially in regions with rice-dominated diets.

Phytoalexins are secondary metabolites that protect the plant against a plethora of diseases [51]. Besides defence against pathogens in the field, post-harvest phytoalexins in the seed may also serve as a natural means to protect the grains against spoilage [52]. Further, phytoalexins have been suggested as beneficial for human health [53]. In our work, we introduce multiple siRNAs possibly regulating biosynthesis of the diterpenoid phytoalexins oryzalectin A-E, momilactone A and phytocassanes (Fig. 6B, Supplementary Table 9).

We also identified 41 embryo-expressed siRNA24 loci possibly targeting ET response and biosynthesis genes (Supplementary Table 10). Seed ET levels have been related to grain size and quality [54, 55] and to drought-induced yield loss [56]. Recent agronomic practices in rice cultivation include a shift from flooded ‘paddy fields’ to aerobic growth conditions for the reduction of greenhouse gas emission and water consumption. The siRNA loci identified here provide a starting point for further research and – if their predicted involvement in ET regulation and/or response could be experimentally confirmed – a new target for rice yield increase under dry conditions.

## Conclusions

This work provides an in-depth analysis of the sRNA profile throughout rice development, adding novel sRNA and tsRNA loci to the existing rice genome annotation. Especially for the economically important, but generally understudied embryo and endosperm tissues, this expanded annotation will be instrumental for future functional research. siRNA24 loci are highly expressed in all tissues and time points under study and are significantly associated with euchromatic TEs, while underrepresented in heterochromatic TEs. Focussing on seed tissues, we found that the embryo contains much less tsRNA than other plant parts, possibly caused by snoRNA-mediated cleavage protection of tRNAs.

Commonly expressed sRNAs between embryo and endosperm are predominantly 24 nt in length and accumulate to higher levels in the endosperm. Analysis of the siRNA21 expression profile uncovered the existence of 21 nt siren in solid stage endosperm tissue, which are generated in an unphased pattern from expressed protein-coding loci or mRNAs. Lastly, through comparison with publicly available datasets we introduce multiple siRNAs possibly involved in the regulation of rice grain yield and quality, which warrant further functional genetic analyses.

## Methods

### Plant material and growth conditions

*Oryza sativa* cultivar Kitaake seeds were germinated in the dark on moist tissue paper for 7 days, before transplant to SAP (sand and absorbent polymer) [57]. These seedlings were grown in a growth chamber at 28 °C under 12 h/12 h light/dark regime and fertilized three times per week with Hoagland solution [58]. At 17 days after transplant to SAP (4–5 leaves stage), in the vegetative growth phase of the rice plant [59], the whole root system and the leaves were sampled. Another set of plants was grown until the generative growth stage. At 6 weeks old, these plants were transferred to a 2:1 SAP:soil mixture and further grown in the greenhouse (28 °C/23 °C day/night temperature, fertilized with Hoagland once per week). They were harvested 3 weeks after emergence of the first flowers (18 week old plants). From these plants, immature endosperm (solid, but not dry), corresponding immature embryos (stages Em9–10: maturation and onset of dormancy) [59], roots and leaf blades were sampled. Three biological replicates (“samples”) were used per condition, each sample consisting of the pooled material from at least 3 plants.

### RNA sequencing and data processing

Total RNA was extracted using the Quick-RNA Plant Miniprep Kit (Zymo Research). cDNA libraries were prepared from 240 ng total RNA by the NXTGNT Ghent University sequencing Facility with the Small RNA-Seq Library Prep Kit (Lexogen, 052) following the manufacturer’s protocol. Size selection was done on gel for 20–80 nt. Since these are small fragments, there was no ribodepletion. The cDNA was sequenced on Illumina NextSeq500 in one run, single-end, read length 76 nt. Adapter sequences and low-quality reads were removed using Trimmomatic (v. 0.38) [60] with default settings, requiring a minimum length of 16 nt. Quality control was performed with FastQC (v. 0.11.8) before and after trimming. Subsequently, reads were aligned to the rice genome (IRGSP 1.0) using ShortStack (v. 3.8.5) align_only mode with default parameters (U mode for multimapping reads) [25, 27]. Nipponbare-based annotation was used for this, since the genome of the Kitaake and Nipponbare cultivars is very similar [61] and the Nipponbare genome is better annotated. After quality control, one vegetative leaf sample of insufficient quality had to be removed from the analysis.

### Identification of novel sRNAs

Aligned reads were removed if they originated from ribosomal RNA, tRNA, small nuclear RNA or small nucleolar RNA regions annotated in Ensembl Plants (v. 47) [62] (50% overlap with the gene required). Additionally, known sRNA loci (recently identified by [23] and [21]) were discarded (100% overlap required). Reads were then pooled across all biological replicates and tissues. On this data, new sRNA loci were predicted with ShortStack clustering, requiring a minimum coverage of 0.5 reads per million mapped (7 reads with the current dataset) [25]. All other parameters were set to their default values. Identified clusters were filtered further by discarding those overlapping with known sRNA loci [21, 23] and selecting those which were expressed (FPKM > 2) in at least two samples. ‘sRNA loci’ refers to genomic regions giving rise to a set of sRNAs, probably originating from the same precursor [25].

### sRNA expression analysis

Novel sRNAs were combined with the sRNAs already annotated by Lunardon et al. (2020) [21] and Liu et al. (2017) [23]. For overlapping annotations, Lunardon et al. (2020) [21] was preferred over Liu et al. (2017) [23] as the former annotation is based on a more substantial dataset of 128 libraries. Count tables of these annotated and newly predicted sRNA genes were made using the GenomicAlignments package from R Bioconductor [63]. To take into account both library size and the varying length of sRNA clusters, counts were normalized as FPKM (fragments per kilobase per million reads mapped) and genes were considered expressed when all biological replicates had an FPKM > 2. Principal component analysis and analysis for differential expression were carried out on the fragment count data with DESeq2 using FDR threshold alpha = 0.05 [64]. Where needed, the threshold for independent filtering of the results, theta, was adapted manually to enable finding the optimal filtering cut-off.

### Association with transposable elements and coding genes

The Bioconductor regioneR package [65] (1000 permutations, significance level alpha = 0.05) was used to assess significance of overlap between genomic regions. For these analyses, TE regions were obtained from the Rice TE database (RiTE v. 1.0) [66] and protein-coding genes from IRGSP 1.0.42. BEDTools was used to extract 1 kb upstream (from transcription start site) and 1 kb downstream (from transcription end site) regions.

To evaluate overlap of sRNAs expressed in embryo and endosperm with protein-coding genes in the corresponding tissues, raw mRNA-seq fastq files were obtained for 1 embryo and 2 endosperm samples from SRA, accessions SRR352204, SRR352206 and SRR352209, respectively [33]. Adapter sequences and low-quality reads were removed using Trimmomatic (v. 0.38) [60] with default settings, requiring a minimum length of 20 nt. Quality control was performed with FastQC (v. 0.11.8) before and after trimming. Subsequently, reads were aligned to the rice genome (IRGSP 1.0) using bowtie2 (v. 2.3.4.3) in end-to-end mode [67, 68]. Count tables were generated with the GenomicAlignments package [63]. Genes were divided into highly expressed (average FPKM > 10), medium expressed (2 < FPKM <= 10), lowly expressed (0 < FPKM <= 2) or not expressed (FPKM = 0).

For functional prediction of embryo and endosperm siRNAs, protein-coding genes were selected if they overlapped with expressed siRNA24 in their 1 kb upstream, gene body or 1 kb downstream regions. These genes were further filtered based in their expression level in embryo or endosperm (FPKM <= 2, see above) and on the level of DNA methylation. DNA methylation data in embryo and endosperm was retrieved from the gene expression omnibus (GEO) (GSE22591) and processed gff files were downloaded [34]. From these files, % methylation of the genome in 1 kb upstream, gene body and 1 kb downstream regions of protein-coding genes was determined as the fraction of methylated cytosines relative to the total number of cytosines in each region. Only when the methylation level of siRNA24-overlapping regions was situated in the upper quantile (> 20% or > 12% for embryo or endosperm, respectively), the corresponding protein-coding gene was selected as a possible siRNA24 target.

For these putative targets, GO enrichment analysis was carried out using Monocots PLAZA (v. 4.5) [35] in default mode with Bonferroni *p*-value correction for multiple testing. Enriched GO terms were summarized with REVIGO [36]. Additionally, these genes were loaded in OryzaCyc (v. 7.0) [37] and MapMan (V.3.6) [69] for functional prediction.

### Study of tRNA-derived sRNAs

tRNA annotations in Ensembl Plants (v. 47) were supplemented with tRNAs from the plantRNA database (http://plantrna.ibmp.cnrs.fr/). The latter are annotated on a previous genome build (msu5). To align them to the latest build (IRGSP1.0/msu7), the tRNA sequences were retrieved including their 50 base pairs (bp) upstream and 25 bp downstream sequences for precision. They were aligned to the rice genome using bowtie2 (v. 2.3.4.3) in end-to-end mode [67, 68]. Only tRNAs with 100% match to the genome were kept. Next, tRNA annotations not overlapping with Ensembl annotations were added to the annotation file. Only tRNAs encoded in nuclear DNA were used.

To identify tsRNA reads, tRNA sequences were retrieved from this combined annotation file using BEDTools. A BLAST database was built from both mature tRNAs (adding the 3′ CCA) and ‘primary’ tRNAs extended with their 40pb upstream and 40 bp downstream regions. From the trimmed sRNA-seq reads, 14-40 nt reads were selected and compared to this tRNA database with NCBI blastn (v. 2.9.0+) in blastn-short mode. For maximum stringency, only reads aligning over their entire length with 100% identity were kept. Reads were further selected and classified based on their mapping position and length as in Gupta et al. (2018) [28]. Only reads exactly starting at the 5′ end of mature tRNAs (tRF-5 or 5tiR), ending at exactly the last base of the mature tRNA (tRF-3 or 3tiR) and reads aligning in the 3′ downstream region (tRF-1) were kept for further analysis.

To determine relative tsRNA expression in each sample, the fraction of tRNA-derived reads among all 14-40 nt reads was normalized to 4 reference sRNA genes. These were selected based on the DESeq2 likelihood ratio test (LRT) as genes that were not differentially expressed between the 6 studied tissues (adjusted *p*-value > 0.5) and still had sufficiently high expression levels (FPKM > 2 for all biological replicates). The fraction of 14-40 nt reads falling within each of these reference genes was calculated, and the average over all 4 genes was taken per sample. tRNA-derived fractions were then normalized to these reference sRNA genes by dividing tRNA fractions by average reference gene fractions per sample. From this, relative expression levels were calculated as the log2 fold change relative to the vegetative phase root.

For comparison to the plant tRF [28] and tsRBase [29] databases, all unique tsRNA sequences were compared to the rice sequences from the database using NCBI blastn (v. 2.9.0+) in blastn-short mode or the website’s built-in BLAST tool, respectively.

### Independent validation of sRNA-seq results

tsRNA expression was validated in independent 17 day old root and leaf samples with reverse transcription PCR (RT-PCR). Three biological replicates were taken, each replicate consisting of pooled tissue of 3 different plants. RNA was extracted with the Quick-RNA Plant Miniprep Kit (Zymo Research). cDNA was prepared on DNAse-treated RNA with the Tetro cDNA Synthesis Kit (Bioline). Stem-loop primers were used for cDNA synthesis to capture the short tsRNA sequences, a method which has been proven successful before to enable detection of tsRNA expression [70]. PCR was carried out with Taq DNA polymerase (VWR Belgium). All primers are shown in Supplementary Table 1.

## Supplementary Information

**Additional file 1.** Supplementary Tables 1–4 in a word document.

**Additional file 2.** Supplementary figures 1–10 in a word document.

**Additional file 3: Supplementary Table 5.** List of identified siren21 loci.

**Additional file 4: Supplementary Table 6.** List of potentially siRNA-regulated genes in the embryo.

**Additional file 5: Supplementary Table 7.** List of potentially siRNA-regulated genes in the endosperm.

**Additional file 6: Supplementary Table 8.** Gene ontology enrichment analysis on siRNA targets in the embryo (Additional file 4). GO enrichment analysis was carried out with the Monocots PLAZA 4.5 workbench [35] in default mode. BP = biological process; CC = cellular compartment; MF = molecular function.

**Additional file 7: Supplementary Table 9.** Potential siRNA24 regulators of diterpenoid biosynthesis in the embryo, as annotated in OryzaCyc (v. 7.0) [37].

**Additional file 8: Supplementary Table 10.** Potential siRNA24 regulators of ethylene levels and response in embryo and endosperm, as annotated in MapMan (V3.6).

**Additional file 9.** Combined annotation file of the novel sRNAs identified with ShortStack (v. 3.8.5) and the identified tRNA-derived sRNAs (tsRNAs) in gff3 format.

**Additional file 10.** Detailed description of each novel sRNA locus identified in this work, including the sequence of the most abundant sRNA (“MajorRNA”) and the length distribution and originating strand of the reads within each locus.

**Additional file 11.** CSV files of differentially expressed sRNAs between the different tissues, of the format x_vs_y.csv, indicating that log2 fold changes are calculated relative to y.

## Data Availability

The datasets generated during the current study are available in the European Nucleotide Archive (ENA) under accession number PRJEB37381 and are available at https://www.ebi.ac.uk/ena/browser/home. Supplementary data for this article can be accessed on the publisher’s website.
