# Correction to: Spatiotemporal expression profle of novel and known small RNAs throughout rice plant development focussing on seed tissues

**DOI:** 10.1186/s12864-022-08381-3

**Published:** 2022-02-16

**Authors:** Anikó Meijer, Tim De Meyer, Klaas Vandepoele, Tina Kyndt

**Affiliations:** 1 Department of Biotechnology, Ghent University, Ghent, Belgium; 2 Department of Data Analysis and Mathematical Modelling, Ghent University, Ghent, Belgium; 3 Department of Plant Biotechnology and Bioinformatics, Ghent University, Ghent, Belgium; 4 VIB Center for Plant Systems Biology, Ghent, Belgium; 5 Bioinformatics Institute Ghent, Ghent University, Ghent, Belgium

**Correction to: BMC Genomics 23, 44 (2022)**

**https://doi.org/10.1186/s12864-021-08264-z**

Following publication of the original article [1], a typographical error was reported in the ‘Background’ section of the article. The incorrect and corrected sentences are given below, with the correction highlighted in bold:

The incorrect sentence read:

Twenty-one nt siRNAs, on the other hand, mainly cause transcriptional repression through the plant-specific RNA-directed DNA methylation (RdDM) pathway, inducing de novo DNA methylation [8].

The corrected sentence is:

**Twenty-four** nt siRNAs, on the other hand, mainly cause transcriptional repression through the plant-specific RNA-directed DNA methylation (RdDM) pathway, inducing de novo DNA methylation [8].

The original article [1] has been corrected.
