# Supplementary material for: Spatiotemporal expression profile of novel and known small RNAs throughout rice plant development focussing on seed tissues
Source: BMC Genomics. 2022 Jan 11;23:44. doi: 10.1186/s12864-021-08264-z (PMC8750796; doi:10.1186/s12864-021-08264-z)
Supplement: Supplementary file 1 — Additional file 1. Supplementary Tables 1–4 in a word document. [file 12864_2021_8264_MOESM1_ESM.docx]

**Supplementary table 1**: List of primers used for RT-PCR validation of tsRNAs. In the stem-loop primers, bold regions indicate the gene-specific nucleotides following the stem-loop.

| **tsRNA** | **stem-loop primer** | **forward primer** | **universal reverse primer** |
| --- | --- | --- | --- |
| orysa-PheGAA.5tiR | GTCGTATCCAGTGCAGGGTCCGAGGTATTCGCACTGGATACGAC**TCAGTC** | GTCAGGATAGCTCAGTTGGTAGAG | GTGCAGGGTCCGAGGT |
| orysa-AlaCGC.tRF5 | GTCGTATCCAGTGCAGGGTCCGAGGTATTCGCACTGGATACGAC**TATGAG** | GGAGCCGGGGACGTAG |  |
| orysa-GluTTC.5tiR | GTCGTATCCAGTGCAGGGTCCGAGGTATTCGCACTGGATACGAC**AGCCAG** | TCCGTTGTCGTCCAGCG |  |
| orysa-ArgACG.tRF5 | GTCGTATCCAGTGCAGGGTCCGAGGTATTCGCACTGGATACGAC**TCCTCT** | GGAACAGGGCCTGTAGCTC |  |

**Supplementary** **Table** 2: Mapping statistics of the sRNA-seq analysis. Veg = vegetative phase; Gen = generative phase; MMAP = multimapping.

| **Sample** | **Tissue** | **raw reads** | **trimmed reads** | **unique mappers** | **MMAP reads** | **total mapped** | **unmapped** | **unique fraction** | **MMAP fraction** | **unmapped fraction** |
| --- | --- | --- | --- | --- | --- | --- | --- | --- | --- | --- |
| A1 | Veg leaf | 8,440,274 | 7,189,265 | 308,648 | 3,420,575 | 3,729,223 | 3,460,042 | 0.043 | 0.476 | 0.481 |
| A2 | Veg leaf | 8,449,328 | 8,085,021 | 2,180,206 | 1,888,377 | 4,068,583 | 4,016,438 | 0.270 | 0.234 | 0.497 |
| A3 | Veg leaf | 6,327,355 | 6,103,472 | 1,714,054 | 1,335,315 | 3,049,369 | 3,054,103 | 0.281 | 0.219 | 0.500 |
| B1 | Veg root | 8,486,223 | 7,678,349 | 1,521,507 | 1,797,763 | 3,319,270 | 4,359,079 | 0.198 | 0.234 | 0.568 |
| B2 | Veg root | 9,158,991 | 8,104,145 | 1,565,800 | 2,321,573 | 3,887,373 | 4,216,772 | 0.193 | 0.286 | 0.520 |
| B3 | Veg root | 7,974,029 | 7,039,496 | 1,326,531 | 1,446,867 | 2,773,398 | 4,266,098 | 0.188 | 0.206 | 0.606 |
| C1 | Gen leaf blade | 9,084,415 | 8,347,271 | 1,843,951 | 2,446,929 | 4,290,880 | 4,056,391 | 0.221 | 0.293 | 0.486 |
| C2 | Gen leaf blade | 10,952,640 | 9,698,084 | 972,930 | 4,653,887 | 5,626,817 | 4,071,267 | 0.100 | 0.480 | 0.420 |
| C3 | Gen leaf blade | 8,436,838 | 7,764,861 | 1,736,114 | 1,960,011 | 3,696,125 | 4,068,736 | 0.224 | 0.252 | 0.524 |
| D1 | Gen root | 10,718,511 | 9,331,294 | 1,368,014 | 2,789,839 | 4,157,853 | 5,173,441 | 0.147 | 0.299 | 0.554 |
| D2 | Gen root | 9,354,072 | 8,352,986 | 1,328,348 | 2,468,019 | 3,796,367 | 4,556,619 | 0.159 | 0.295 | 0.546 |
| D3 | Gen root | 9,470,921 | 8,511,275 | 1,234,291 | 3,244,702 | 4,478,993 | 4,032,282 | 0.145 | 0.381 | 0.474 |
| E1 | Embryo | 9,717,276 | 9,405,560 | 5,860,938 | 1,036,675 | 6,897,613 | 2,507,947 | 0.623 | 0.110 | 0.267 |
| E2 | Embryo | 9,400,538 | 9,035,946 | 5,643,094 | 1,011,285 | 6,654,379 | 2,381,567 | 0.625 | 0.112 | 0.264 |
| E3 | Embryo | 8,831,711 | 8,564,951 | 5,361,817 | 882,896 | 6,244,713 | 2,320,238 | 0.626 | 0.103 | 0.271 |
| F1 | Endosperm | 8,349,277 | 7,061,280 | 1,663,035 | 2,055,524 | 3,718,559 | 3,342,721 | 0.236 | 0.291 | 0.473 |
| F2 | Endosperm | 9,969,133 | 9,150,547 | 2,379,936 | 1,948,244 | 4,328,180 | 4,822,367 | 0.260 | 0.213 | 0.527 |
| F3 | Endosperm | 6,480,382 | 6,038,374 | 1,316,110 | 1,119,154 | 2,435,264 | 3,603,110 | 0.218 | 0.185 | 0.597 |

**Supplementary table 3**:Fraction of all mapped reads that aligned in unannotated regions.

| **Tissue** | **Fraction of unannotated reads** | |
| --- | --- | --- |
|  | **Average** | **sd** |
| Veg root | 0.110 | 0.013 |
| Gen root | 0.082 | 0.011 |
| Veg leaf | 0.079 | 0.013 |
| Gen leaf blade | 0.072 | 0.026 |
| Embryo | 0.230 | 0.024 |
| Endosperm | 0.272 | 0.031 |

**Supplementary table 4:** Expressed miRNAs detected in our study.

| **Expressed in** | **name** | **description** | **family** |
| --- | --- | --- | --- |
| Endosperm | osa-b1.0r1-121120 | MIRNA locus with predominant RNA size of 21 |  |
| Embryo | osa-b1.0r1-60176 | MIRNA locus with predominant RNA size of 20 | miR394 |
| (Embryo, Endosperm) | osa-MIR169o | MIRNA locus with predominant RNA size of 21 | miR169 |
| (Gen.root, Embryo, Endosperm) | osa-b1.0r1-43465 | MIRNA locus with predominant RNA size of 24 |  |
| (Gen.root, Veg.shoot) | osa-b1.0r1-51521 | MIRNA locus with predominant RNA size of 24 |  |
| (Veg.root, Gen.root, Embryo) | osa-b1.0r1-75467 | MIRNA locus with predominant RNA size of 24 |  |
| (Veg.root, Gen.root, Veg.shoot, Gen.shoot) | osa-b1.0r1-73745 | MIRNA locus with predominant RNA size of 24 |  |
| (Veg.root, Gen.root, Veg.shoot, Gen.shoot, Embryo, Endosperm) | osa-b1.0r1-110916 | MIRNA locus with predominant RNA size of 24 |  |
| (Veg.root, Gen.root, Veg.shoot, Gen.shoot, Embryo, Endosperm) | osa-b1.0r1-113911 | MIRNA locus with predominant RNA size of 24 |  |
| (Veg.root, Gen.root, Veg.shoot, Gen.shoot, Embryo, Endosperm) | osa-b1.0r1-137324 | MIRNA locus with predominant RNA size of 24 |  |
| (Veg.root, Gen.root, Veg.shoot, Gen.shoot, Embryo, Endosperm) | osa-b1.0r1-35170 | MIRNA locus with predominant RNA size of 21 | miR1846 |
| (Veg.root, Gen.root, Veg.shoot, Gen.shoot, Embryo, Endosperm) | osa-b1.0r1-75920 | MIRNA locus with predominant RNA size of 24 |  |
