# Supplementary material for: Spatiotemporal expression profile of novel and known small RNAs throughout rice plant development focussing on seed tissues
Source: BMC Genomics. 2022 Jan 11;23:44. doi: 10.1186/s12864-021-08264-z (PMC8750796; doi:10.1186/s12864-021-08264-z)
Supplement: Supplementary file 2 — Additional file 2. Supplementary figures 1–10 in a word document. [file 12864_2021_8264_MOESM2_ESM.docx]

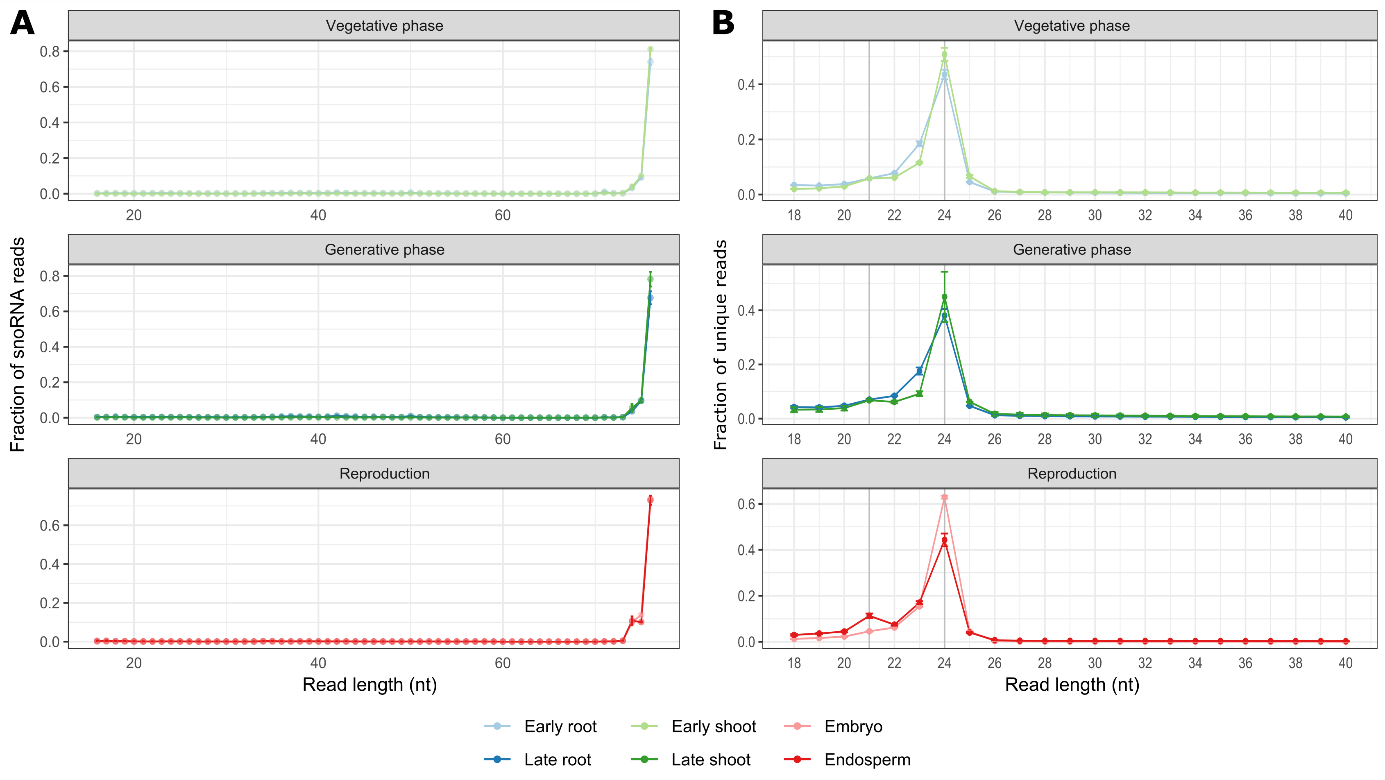


**Supplementary Figure 1**: **A)** Size distribution of reads mapping to snoRNA loci, for each of the studied tissues. **B)** Size distribution of the unique RNA reads for each of the tissues. In all panels, error bars indicate the standard deviation. Early = vegetative phase; late = vegetative phase. Early shoot indicates leaves and late shoot indicates leaf blades.


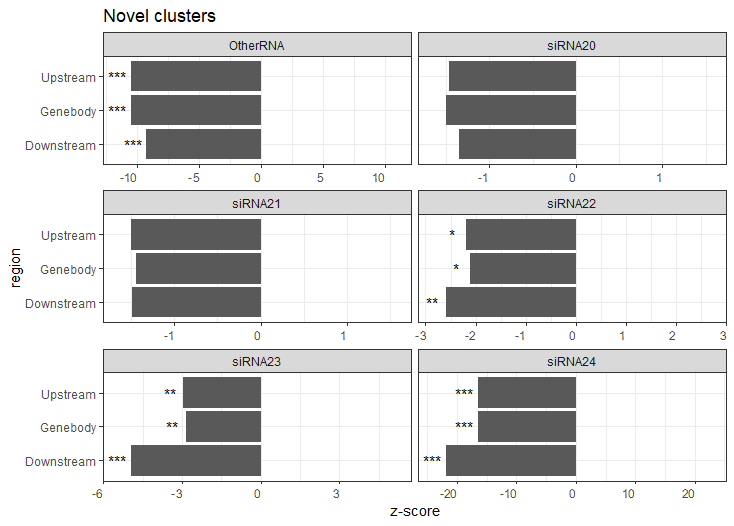


**Supplementary Figure 2**: Association of the different types of novel sRNA loci with annotated protein-coding genes in their 1kb upstream, gene body or 1kb downstream regions. Permutation tests with 1000 permutations were performed to estimate the null distribution of sRNA loci randomly overlapping with the regions of interest, using regioneR. z-scores were calculated as the deviation of the actual number of overlaps from the number of overlaps under the null distribution, relative to the standard deviation. Stars indicate significant enrichment/depletion * = p < 0.05; ** = p < 0.01; *** = p < 0.001.


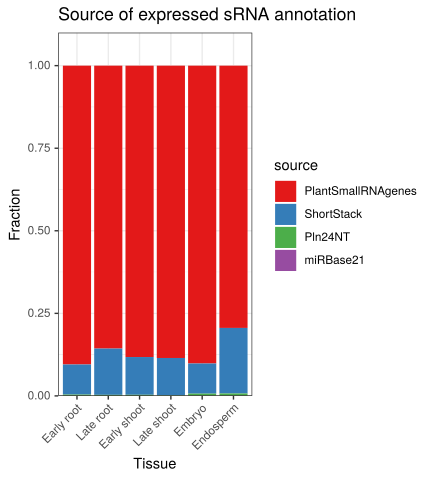


**Supplementary** **Figure** **3**: Source of the sRNA genes which were found to be expressed in the different tissues (FPKM > 2 in all biological replicates). PlantSmallRNAgenes = PlantSmallRNA database (Lunardon et al., 2020)*;* Pln24NT = pln24NT database (Liu et al., 2017); miRBase21 = miRBase version 21 (Kozomara & Griffiths-Jones, 2014; Lunardon et al., 2020); ShortStack = novel loci discovered in this study. Early = vegetative phase; late = generative phase. Early shoot indicates leaves and late shoot indicates leaf blades.


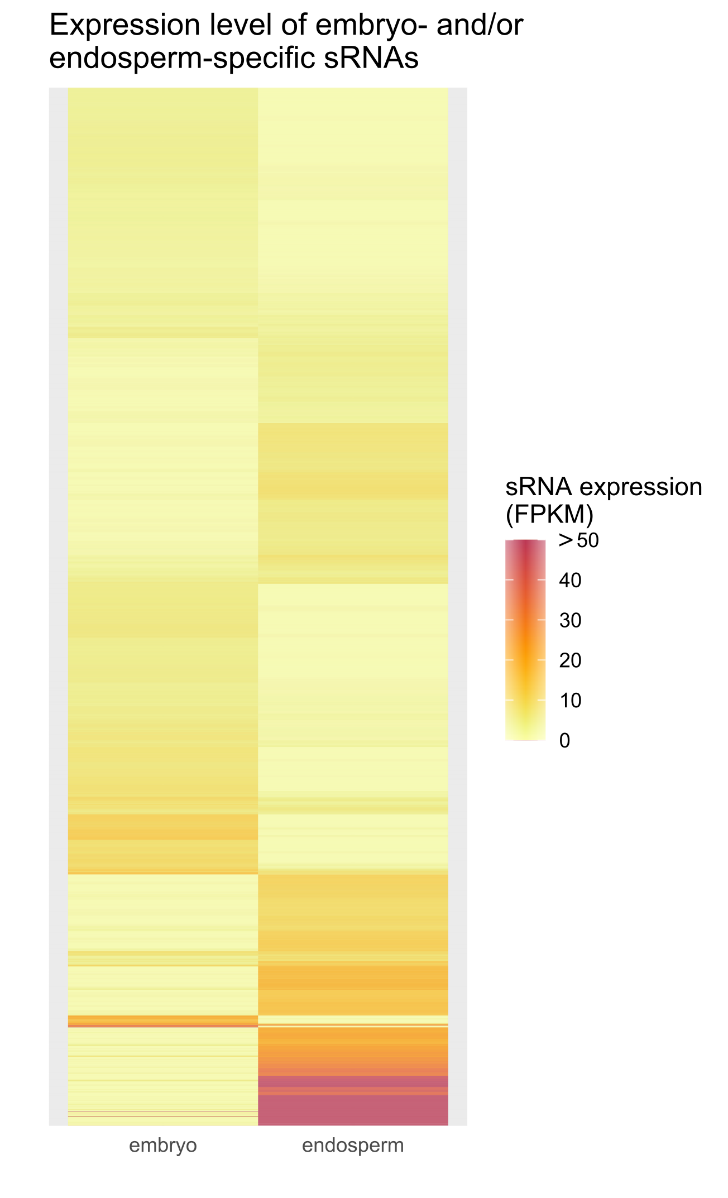


**Supplementary figure 4:** Expression level (FPKM) of sRNAs commonly expressed in embryo and endosperm, but with read count of 0 in all non-seed tissues.


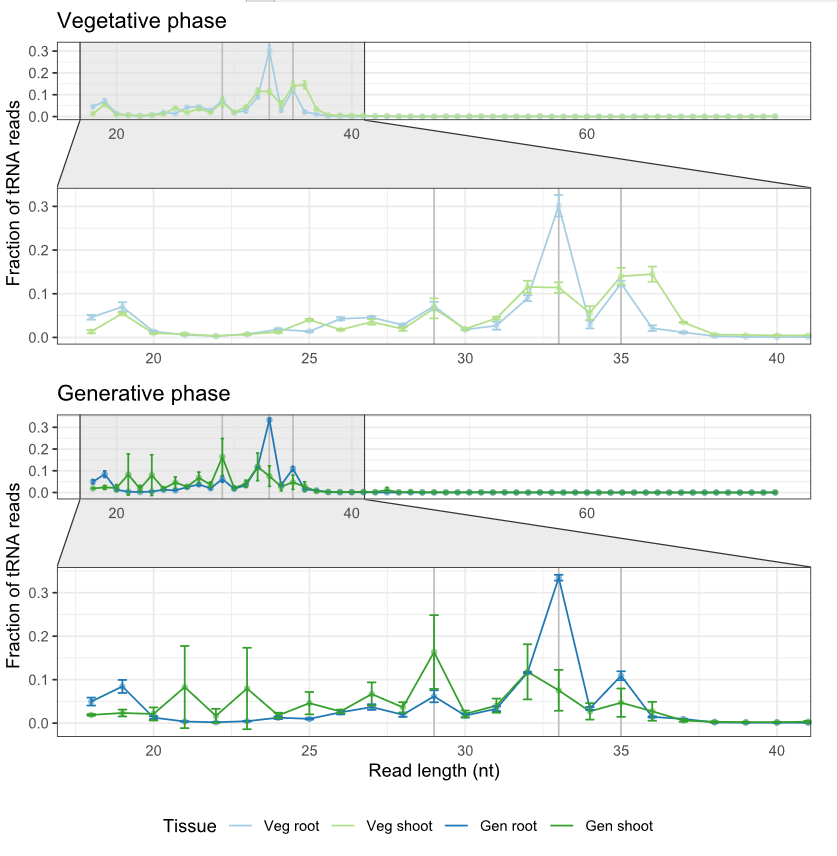


**Supplementary figure 5**: Size distribution of tRNA-derived reads in roots and leaves at the vegetative phase and roots and leaf blades at the generative phase.


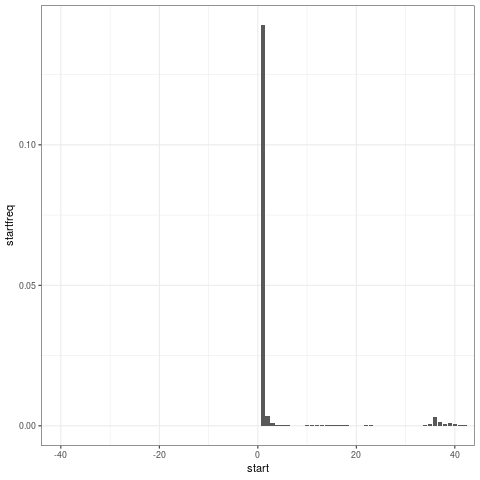

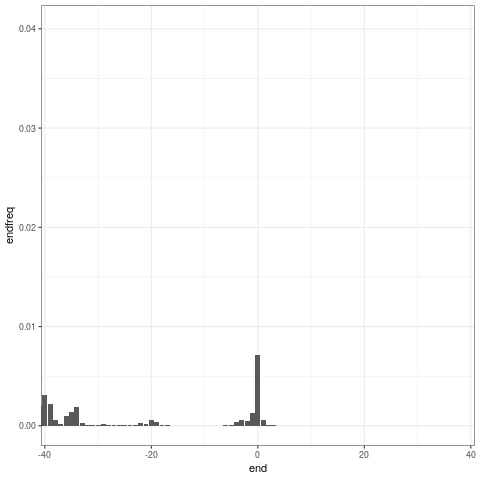


**Supplementary figure 6**: Mapping of the 5’ (left) and 3’ (right) end of tRNA-derived reads on mature tRNA transcripts. Distributions are shown of the distance between read start/end and transcription start/end of the corresponding tRNA.


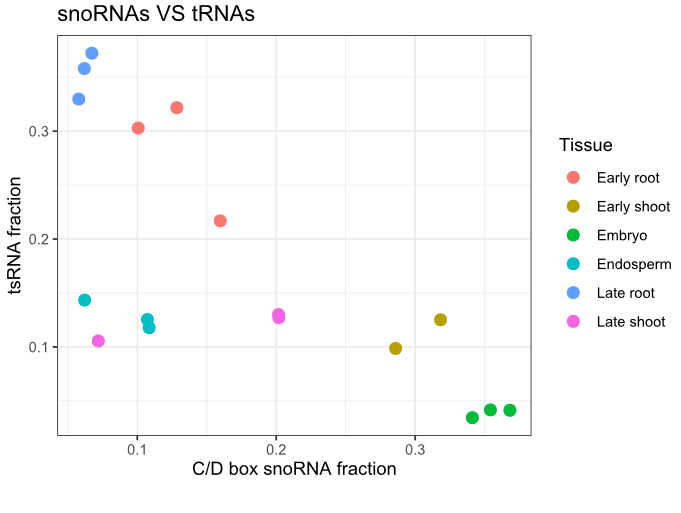


**Supplementary figure 7**: Fraction of C/D box snoRNAs (compared to all mapped reads) vs fraction of tsRNAs (compared to number of 14-40nt reads). Early = vegetative phase; late = generative phase. Early shoot indicates leaves and late shoot indicates leaf blades.

**
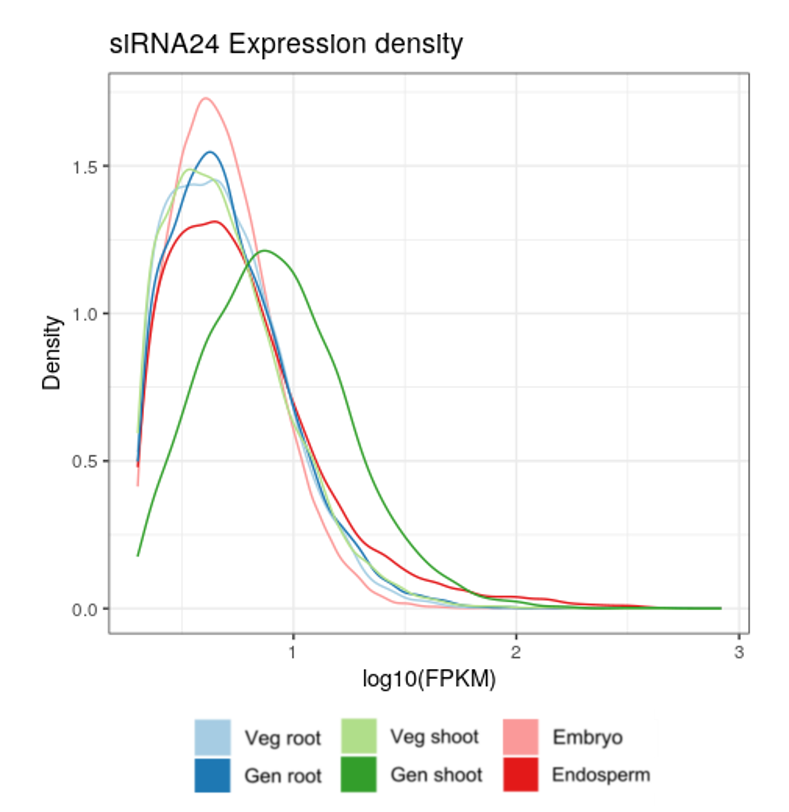
**

**Supplementary figure 8:** Expression density of expressed siRNA24 loci in all tissues


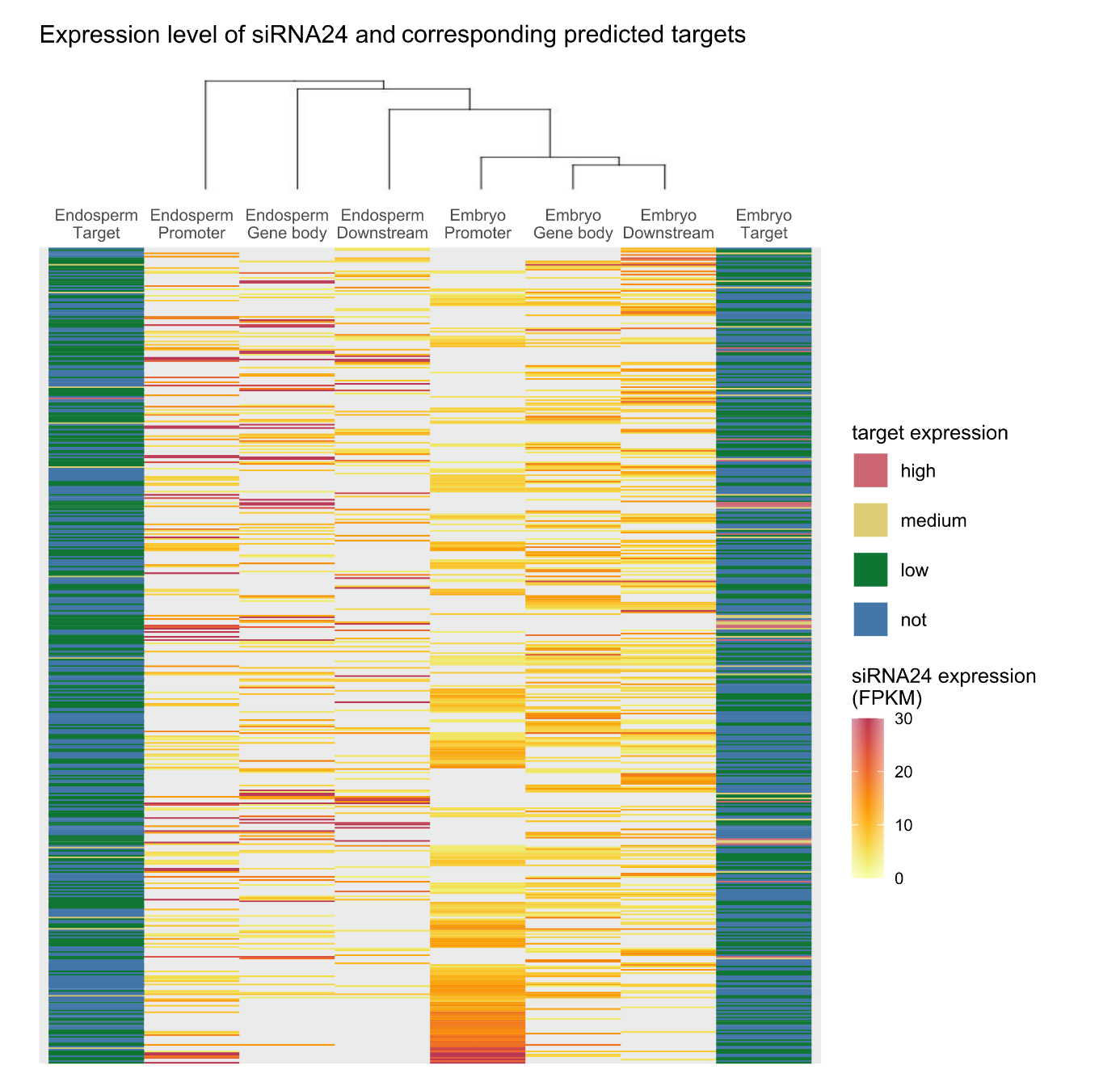


**Supplementary figure 9:** Expression levels of predicted siRNA24 targets in embryo and endosperm (blue to red bars), and their corresponding siRNA24 in each of the 3 studied regions (yellow to red). Expression of predicted targets is classified as in figure 6: high (FPKM > 10), medium (FPKM between 10 and 2), low (FPKM between 2 and 0) or not expressed (FPKM = 0).


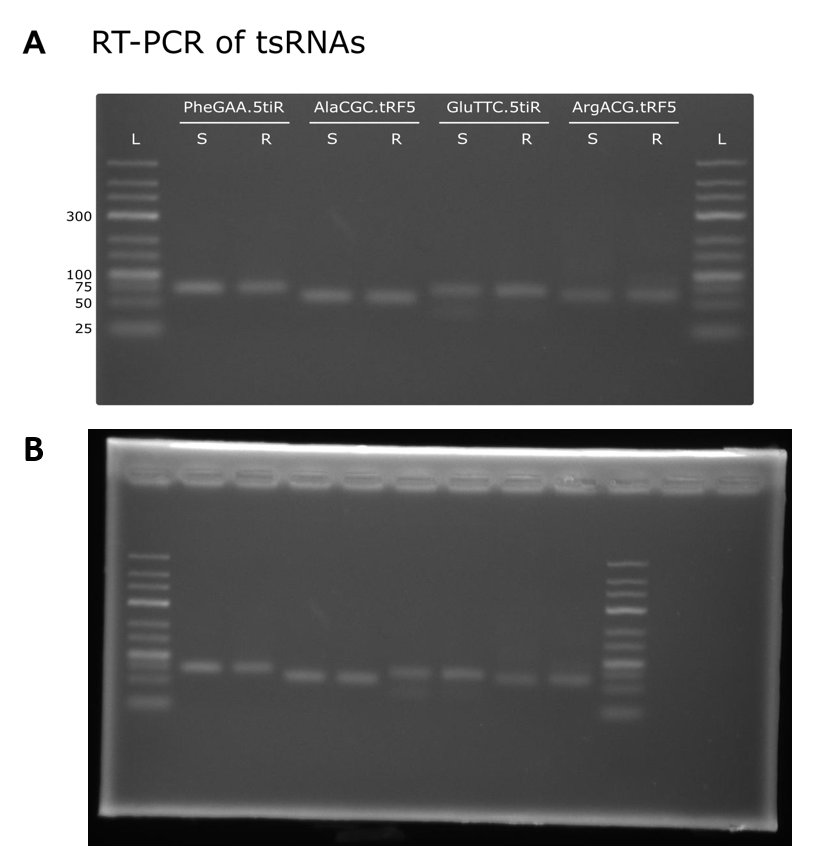


**Supplementary figure 10:** RT-PCR results confirming expression of selected tsRNA genes in leaves (shoot, S) and roots (R) at the vegetative stage. L = gene ruler low range DNA ladder (Thermo Scientific). Numbers indicate the fragment sizes of the ladder bands. Given the 44bp stem-loop that was added to the fragments, sizes of 79, 60, 77 and 63bp were expected, respectively from left to right. Primers are shown in Supplementary table 1. The uncropped picture of the electrophoresis gel is shown in panel B.
